# Construction and Validation of a Necroptosis-Related lncRNA Signature in Prognosis and Immune Microenvironment for Glioma

**DOI:** 10.1155/2022/5681206

**Published:** 2022-08-27

**Authors:** Fan Jiang, Zheng Zhan, Yanbo Yang, Guangjie Liu, Song Liu, Jingyu Gu, Zhouqing Chen, Zhong Wang, Gang Chen

**Affiliations:** ^1^Department of Neurosurgery & Brain and Nerve Research Laboratory, The First Affiliated Hospital of Soochow University, Suzhou 215006, Jiangsu, China; ^2^Department of Neurosurgery, China-Japan Friendship Hospital, Beijing, China; ^3^Department of General Surgery, Dushu Lake Hospital Affiliated to Soochow University, Suzhou 215006, Jiangsu, China

## Abstract

**Background:**

Glioma is the most common primary brain tumor, representing approximately 80.8% of malignant tumors. Necroptosis triggers and enhances antitumor immunity and is expected to be a new target for tumor immunotherapy. The effectiveness of necroptosis-related lncRNAs as potential therapeutic targets for glioma has not been elucidated.

**Methods:**

We acquired RNA-seq data sets from LGG and GBM samples, and the corresponding clinical characteristic information is from TCGA. Normal brain tissue data is from GTEX. Based on TCGA and GTEx, we used univariate Cox regression to sort out survival-related lncRNAs. Lasso regression models were then built. Then, we performed a separate Kaplan-Meier analysis of the lncRNAs used for modeling. We validated different risk groups via OS, DFS, enrichment analysis, comprehensive immune analysis, and drug sensitivity.

**Results:**

We constructed a 12 prognostic lncRNAs model after bioinformatic analysis. Subsequently, the risk score of every glioma patient was calculated based on correlation coefficients and expression levels, and the patients were split into low- and high-risk groups according to the median value of the risk score. A nomogram was established for every glioma patient to predict prognosis. Besides, we found significant differences in OS, DFS, immune infiltration and checkpoints, and immune therapy between different risk subgroups.

**Conclusion:**

Predictive models of 12 necroptosis-related lncRNAs can facilitate the assessment of the prognosis and molecular characteristics of glioma patients and improve treatment modalities.

## 1. Introduction

Glioma is the most common primary brain tumor, representing approximately 80.8% of malignant tumors [1]. Current research has made significant progress in the treatment of glioma surgery, radiotherapy, and chemotherapy. Still, the limitations of the current therapy of glioma, including the impact on patients' neurological function, inferior quality of life, and heavy burden on patients' families, cannot be ignored [2]. Although immunotherapy has made considerable progress as a new treatment for malignancies, the 5-year overall survival (OS) for glioma remains below 35% without significant improvement [1]. It urges us to explore more precise and tolerable therapies for glioma.

The utility of necroptosis in cancer is complex. On the one hand, the expression of key regulators in the necroptosis pathway is generally downregulated in cancer cells, indicating that cancer cells may escape necroptosis and survive. On the other hand, the expression levels of key regulators are instead elevated in certain types of cancer. Necroptosis has been reported to induce an inflammatory response, promote cancer metastasis, and produce an immunosuppressive tumor microenvironment [3, 4]. Necroptosis has also been found to play a crucial role in neuroinflammation and degenerative lesions of the central nervous system (CNS). Necroptosis can cause a vigorous inflammatory reaction that can dramatically alter the local tissue environment and mediate the pathogenesis of CNS disease [5]. As a form of programmed death that overcomes resistance to apoptosis, necroptosis triggers and enhances antitumor immunity and is expected to be a new target for tumor immunotherapy.

Long noncoding RNA (lncRNA) is a class of RNA molecules with a transcription length of over 200 nt. They do not encode proteins but participate in protein-coding gene regulation in the form of RNA. LncRNA plays an important role in dose compensation effect, epigenetic regulation, cell cycle regulation, and cell differentiation regulation [6]. Previous studies have shown that the p53-inducible lncRNA TRINGS protects cancer cells from necroptosis induced by glucose starvation [7]. This indicates that there is a relationship between lncRNA and necroptosis. Recent research has shown that lncRNA plays an integral role in glioma proliferation, angiogenesis, stem cells, and drug resistance [8]. LncRNA regulates the malignant phenotype of glioma. LncRNA can act as a molecular signaling mediator, regulating the expression of specific genes and corresponding signaling pathways, such as CRNDE-mTOR signaling [9] and the TALC-cMet pathway [10]. Most of the glioma-related lncRNAs serve as “miRNA sponges” to inhibit miRNA activity (e.g., miR-128-3p/GREM1 [11], miR-619-5p/CUEDC2 [12], miR-494-3p/PRMT1 [13], and miR-106b-5p/TUSC2 [14]. This suggests that the function of lncRNA cannot be negligible in glioma. Necroptosis-related lncRNA has also been found to have prognostic value and a correlation with prognosis and therapeutic targets, and immune analysis in a variety of tumors, for instance, gastric cancer [15], stomach adenocarcinoma [16], breast cancer [17], and lung adenocarcinoma [18]. Therefore, our exploration of the function of necroptosis-associated lncRNA in glioma is of significance.

The effectiveness of necroptosis-related lncRNAs as potential therapeutic targets for glioma has not been elucidated. Studies should be made to figure out the relation between them to provide new ideas for molecular biology diagnosis and treatment targets.

## 2. Materials and Methods

### 2.1. Datasets for Glioma Patients

We acquired RNA-seq data sets (HTSeq—Counts and HTSeq—FPKM) of Lower Grade Glioma (LGG) and Glioblastoma Multiforme (GBM) samples, and the corresponding clinical characteristic information is from The Cancer Genome Atlas (TCGA, https://portal.gdc.cancer.gov/). Normal brain tissue data is from Genotype-Tissue Expression Project (GTEX, https://www.gtexportal.org/home/index.html) database. TCGA data is downloaded for the training group. We use R (4.1.2) software and data. table, dplyr, and tidyr R packages to synthesize the data matrix and perform the analysis.

### 2.2. Acquisition of Necroptosis-Related Genes and lncRNAs

We combined the published literature, the Gene Set Enrichment Analysis (GSEA, https://www.gsea-msigdb.org/gsea/index.jsp) data, and the use of the KEGGREST R package to download all genes of the necroptosis pathway on KEGG (https://www.genome.jp/kegg/) to obtain 159 necroptosis-related genes (supplementary materials). We then used the limma R package to identify differentially expressed lncRNAs (Log2 fold change (FC) > 1, false discovery rate (FDR) < 0.05). The selected differentially expressed mRNAs were then subjected to KEGG and GO analysis to explore their functional clustering. Correlation analysis of these differential lncRNAs and necroptosis-related genes has proceeded, yielding necroptosis-associated lncRNA with Pearson correlation coefficients >0.5 and *p* < 0.001.

### 2.3. Establishment and Validation of Prognostic Model

Based on TCGA and GTEx, we used univariate Cox proportional hazards regression analysis to sort out survival-related lncRNAs in necroptosis-related lncRNAs (*p* < 0.05). Lasso regression models were then built using survival-related lncRNAs, and 1000 iterations were performed to acquire a robust model. We select lncRNAs related to progression based on the penalty parameter (*λ*). We performed a separate Kaplan-Meier analysis of the lncRNAs used for modeling. The screened lncRNAs were used for the multivariate Cox regression model. Risk scores were calculated for the prognostic models. We used the following formula to calculate the risk score:

(1) $$\begin{array}{c} \text{Risk score}=\sum_{k=1}^{n}\text{coef}\left({\text{IncRNA}}^{k}\right)*\exp\left({\text{IncRNA}}^{k}\right), \end{array}$$

where the coef (lncRNA^k^) was the short form of the coefficient of lncRNAs correlated with survival in the Cox model and exp (lncRNA^k^) was the expression of lncRNAs. And then, high- and low-risk groups are established based on the median risk score. To assess the significance of the prognostic model, we used the Kaplan-Meier method to generate survival curves for overall survival (OS) and disease-free survival (DFS). We then combined clinical information using age (≥65, <65), gender (male, female), tumor grade (II, III, IV), IDH status (mutation, mild), MGMT status, and risk score to generate univariate and multivariate forest plots and heat maps for determining the applicability of the prognostic model to the clinic. Then, the receiver operating curves (ROC) of 1, 3, and 5 years were used to test the predictive ability of the prognostic model (‘survivalROC'package).

### 2.4. Nomogram and Calibration

The age, gender, tumor grade, IDH status, MGMT status, and risk score were used to set up the nomogram. The Hosmer–Lemeshow test was used to generate correction curves to test whether the predicted results matched the actual.

### 2.5. Gene Set Enrichment Analysis

We used GSEA software and the KEGG gene set to select significantly enriched pathways in high- and low-risk groups. The screening criteria were *p* < 0.05 and FDR < 0.05.

### 2.6. Immune Infiltration Analysis and Immune Checkpoints

To explore the relationship between the prognostic model and the immune microenvironment features, we calculated the immune infiltration statuses between the different groups with the application of TIMER (https://timer.cistrome.org/). Wilcoxon signed-rank test, limma, scales, ggplot2, ggpubr, and ggtext R packages were performed to analyze differences in immune infiltrating cells between high- and low-risk groups. In addition, we made comparisons about TME scores and immune checkpoint activation between low- and high-risk groups by the ggpubr R package. Besides, the immune and stromal scores were analyzed between two different risk subgroups by the ESTIMATE R package. In addition, tumor immune dysfunction and exclusion (TIDE) (https://tide.dfci.harvard.edu) indicates that a higher score corresponds to worse immunotherapy.

### 2.7. Investigation of Drug Sensitivity

We explored the correlation between 138 kinds of drugs and the subgroups identified with prognostic signature genes using the “pRRophetic” package in R to explore the therapeutic response of necroptosis-related lncRNAs, with their drug sensitivity determined by the half-maximal inhibitory concentration (IC50) of glioma patients.

### 2.8. Statistical Analysis

The R software 4.12 and its corresponding packages were utilized for statistical analyses.

## 3. Results

### 3.1. Identification of Necroptosis-Related lncRNAs in Patients with Glioma

**Figure 1:**
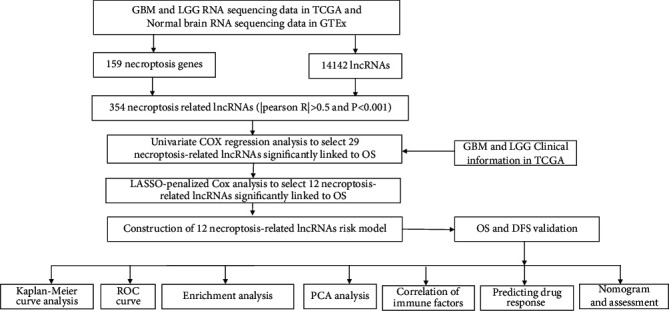
Flowchart of this study.

**Figure 2:**
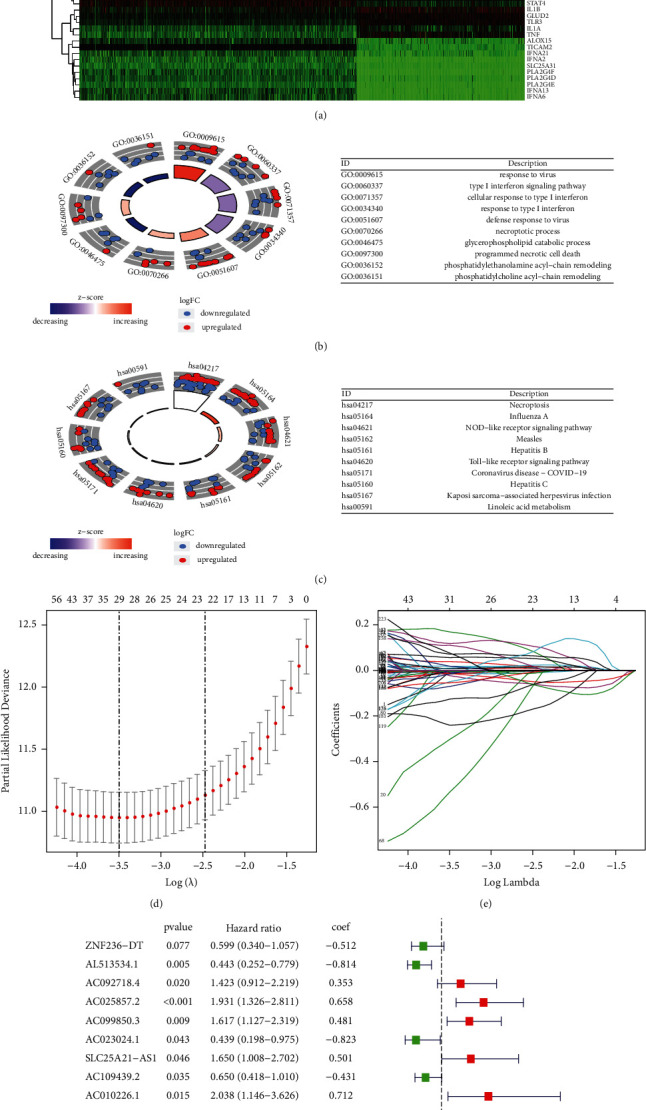
Steps of constructing the prognostic lncRNAs. (a) The differential analysis between glioma (including GBM and LGG patients) and normal brains. (b)-(c) Enrichment analysis of identified differentially expressed mRNA. (d) The lambda plot of necroptosis-related LncRNA by LASSO regression. (e) The LASSO coefficient profiles. (f) Forrest of 12 established LncRNA after multivariate Cox regression analysis.

The detailed process is shown in Figure 1. From TCGA and GTEx matrix, we obtained 1152 normal samples and 667 tumor samples. According to the expression of 159 necroptosis-related genes (Table 1) and 48 differentially expressed mRNAs between normal and tumor samples (Figure 2(a)), GO results showed that differentially expressed necroptosis-associated mRNAs are mainly clustered in response to the virus, type 1 interferon signaling pathway, endosomal membrane, and cytokine receptor binding (Figure 2(b)). KEGG pathway analysis revealed that mRNAs were mainly enriched in necroptosis, influenza A, NOD-like receptors, COVID-19, and hepatitis B and C signaling pathways (Figure 2(c)). We finally got 354 necroptosis-related lncRNAs (Pearson correlation coefficients >0.5 and *p* < 0.001), including 32 downregulated lncRNAs and 322 upregulated lncRNAs. The correlation between necroptosis genes and necroptosis-related lncRNAs is shown in Table S1. These will contribute to investigating the role and mechanisms of necroptosis-related lncRNAs in glioma and other related diseases.

### 3.2. Construction of a Prognostic Model according to Necroptosis-Related lncRNAs in Glioma Patients

Using the univariate Cox regression analysis, we screened 225 necroptosis-related prognostic lncRNAs (Figure S1), which were significantly correlated with OS from 354 necroptosis-related lncRNAs in the whole TCGA set (Table S1). To avoid overfitting and improve the accuracy of the prognostic signature, we performed the LASSO-penalized Cox analysis on these lncRNAs. We acquired 29 lncRNAs related to necroptosis in glioma when the first-rank value of Log(*λ*) was the minimum likelihood of deviance bias (Figures 2(d)-2(e)). Finally, 12 lncRNAs were identified after multivariate Cox regression (Figure 2(f)), and seven lncRNAs were regulated positively by necroptosis genes. Subsequently, the risk score of every glioma patient was calculated based on correlation coefficients calculated, and the patients were split into low- and high-risk groups according to the median value of the risk score. The risk score was calculated as follows:

(2) $$\begin{array}{c} \text{risk score}=\left(0.712*\text{AC}010226.1\exp.\right)+\left(0.6578*\text{AC}025857.2\exp.\right)+\left(0.529*\text{POLR}2\text{J}4\exp.\right)\\ +\left(0.501*\text{SLC}25\text{A}21-\text{AS}1\exp.\right)+\left(0.480*\text{AC}099850.3\exp.\right)+\left(0.352*\text{AC}092718.4\exp.\right)\\ +\left(0.302*\text{AL}590094.1\exp.\right)+\left(-0.431*\text{AC}109439.2\exp.\right)+\left(-0.489*\text{AC}083864.2\exp.\right)\\ +\left(-0.511*\text{ZNF}236-\text{DT }\exp.\right)+\left(-0.814*\text{AL}513534.1\exp.\right)+\left(-0.823*\text{AC}023024.1\exp.\right). \end{array}$$

**Figure 3:**
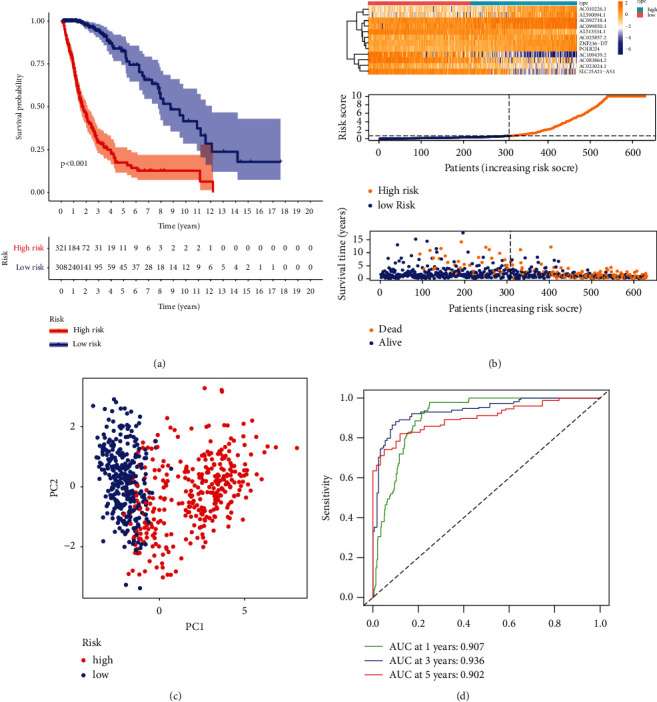
Survival analysis of high- and low-risk patients and calculation of AUC values for such scoring methods. (a) Kaplan-Meier curve indicates the significant difference between the two subgroups. (b) Demonstration of survival status for patients with different scores. (c) PCA analysis. (d) AUCs for this risk score method at 1, 3, and 5 years.

The survival status and survival time of patients in the two different risk groups are shown in Figures 3(a)-3(b). The individual expression of the 12 prognostic necroptosis-related lncRNAs for each patient is shown in Figure S2. The survival analysis shows that the low-risk group has longer OS than the high-risk group. There was a statistical difference in the survival curve between the low-risk and high-risk groups (*p* < 0.001). In addition, we performed PCA on the entire gene expression profiles, 159 necroptosis genes, 354 necroptosis-related lncRNAs, and a risk model classified by the 12 necroptosis-related lncRNAs to detect differences between high and low-risk groups. According to the results of the risk model, there is a discrepancy in the distribution of low- and high-risk groups (Figure 3(c)). These results indicate that the prognostic model can distinguish between the low- and high-risk groups. In addition, the AUCs were 0.907, 0.936, and 0.902 for 1, 3, and 5 years for the risk score (Figure 3(d)).

### 3.3. Assessment of the Necroptosis-Related lncRNA Model and Clinical Features of Glioma Patients

**Figure 4:**
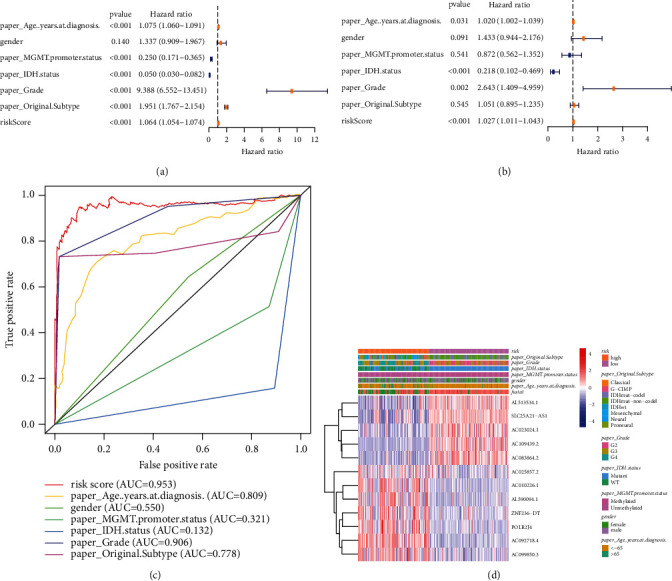
Univariate and multivariate Cox regression analysis for risk score with other clinical features. (a) Forrest plot of univariate Cox regression. (b) Forrest plot of multivariate Cox regression analysis. (c) The ROC curves of risk score and clinicopathological characteristics. (d) Heatmap of patients of different scores and other variables.

To determine whether the predictive signature is independent prognostic factors for patients with glioma, Cox regression analysis was performed on the entire set. Univariate Cox regression analysis showed that age, grade, and risk score were notably associated with the OS in glioma patients. The HR of the risk score and 95% confidence interval (CI) were 1.064 and 1.054–1.074

(*p* < 0.001, Figure 4(a)). Multivariate Cox regression analysis (Figure 4(b)) also showed that age, IDH status, grading, and risk score were significantly associated with the OS in glioma patients. The results of IDH status were contrary to the age, grading, and risk score. The HR of risk score was 1.027, and the 95% CI was 1.011–1.043 (*p* < 0.001). To identify false positives, we also performed ROC analysis for clinical features and the risk score. The AUC of the risk score was also higher than the AUCs of other clinicopathological characteristics, showing that the prognostic risk model was relatively reliable (Figure 4(c)). Heatmaps have valuable data visualization capabilities. We plotted a heatmap for age, gender, grade, risk, survival station, and other common clinicopathological features, describing the overall distribution of clinical information and lncRNA expression in 667 patients in TCGA (Figure 4(d)). Besides, we also explored the differences between high-risk and low-risk patients in different clinicopathological subtypes (Figure S3).

### 3.4. Validation of the Prognostic Model for OS and DFS in TCGA

**Figure 5:**
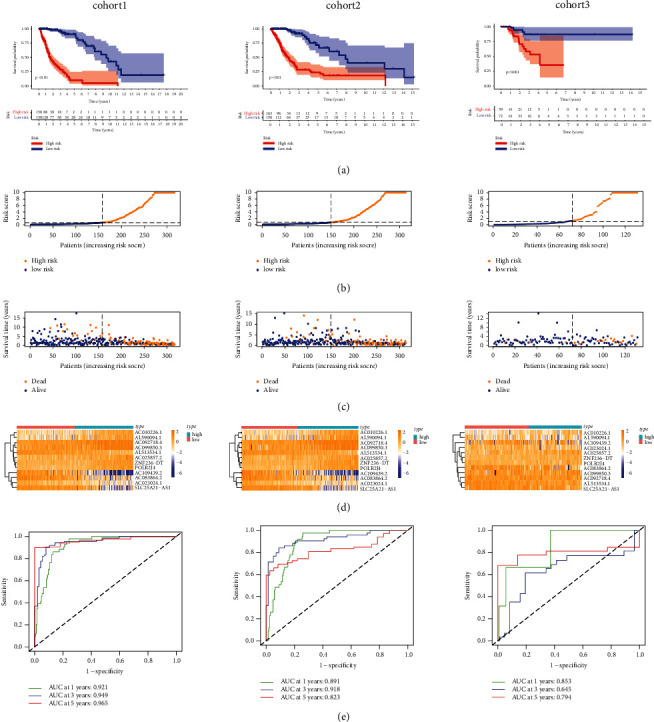
Validation of OS and DFS in survival analysis, distribution, expressional difference, and sensitivity between high-risk and low-risk patients in TCGA. (a) Survival analysis of the 3 cohorts. (b) The risk scores for individuals. (c) The distributions of survival time and patients for risk scores. (d) Heatmap of prognostic necroptosis-related lncRNAs expression in different risk groups.

To test the predictive competence of the prognostic model, we used the uniform formula to calculate risk scores for every patient in TCGA for overall survival and disease-free survival. We randomly and equally divided all glioma patients in the study into cohort 1 and cohort 2. Besides, we downloaded DFS information from cbioportal (https://www.cbioportal.org/) for a portion of glioma patients (*n* = 131) in TCGA. We divided these patients into high- and low-risk groups using the calculations of the previous model and denoted all patients as cohort 3. Figures 5(a)–5(d) depict the distribution of risk grades, the pattern of survival status and survival time, and the expression of the necroptosis-related lncRNAs in TCGA regarding overall survival and disease-free survival. Survival analysis (Figure 5(e)) showed that it is consistent with the results of the TCGA training set. Significant differences display between low- and high-risk groups. The low-risk group has a longer OS than the high-risk group. To test the sensitivity and specificity of the predictive model, we used time-dependent receiver operating characteristics (ROC) along with the area under the ROC curve (AUC) to determine the outcome. As shown in Figure 5(f), the 1-, 3-, and 5-year AUCs of the TCGA cohorts 1 and 2 were 0.824, 0.943, and 0.956. Similarly, the AUCs for DFS were 0.853, 0.645, and 0.794, respectively. This suggests that our prognostic model is approaching clinical reality in terms of OS and DFS.

### 3.5. Construction and Calibration of the Nomogram

**Figure 6:**
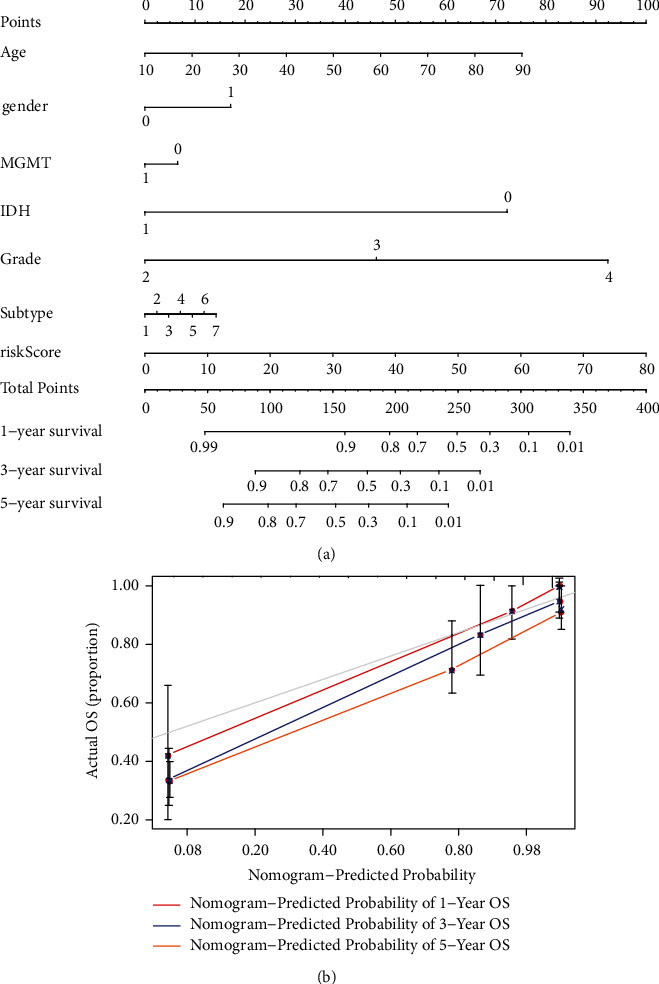
The nomogram predicts the prognosis by calculating risk scores and other clinical features. (a) A nomogram combining identified clinicopathological variables and risk scores predicts survival status for glioma patients. (b) The calibration curves were performed to test the actual survival rates with theoretical results.

We predicted the prognostic model's 1-, 3- and 5-year OS probability by constructing a nomogram containing risk classes and clinical risk factors. Based on clinical characteristics, including age, gender, MGMT methylation, IDH status, WHO grade, subtype, and risk score, the nomogram was established (Figure 6(a)). Additionally, the OS and model prediction rates for years 1, 3, and 5 achieve satisfactory agreement in the calibration curves for TCGA glioma patients (Figure 6(b)).

### 3.6. Investigation of the Immune Factors Based on Prognostic Models

**Figure 7:**
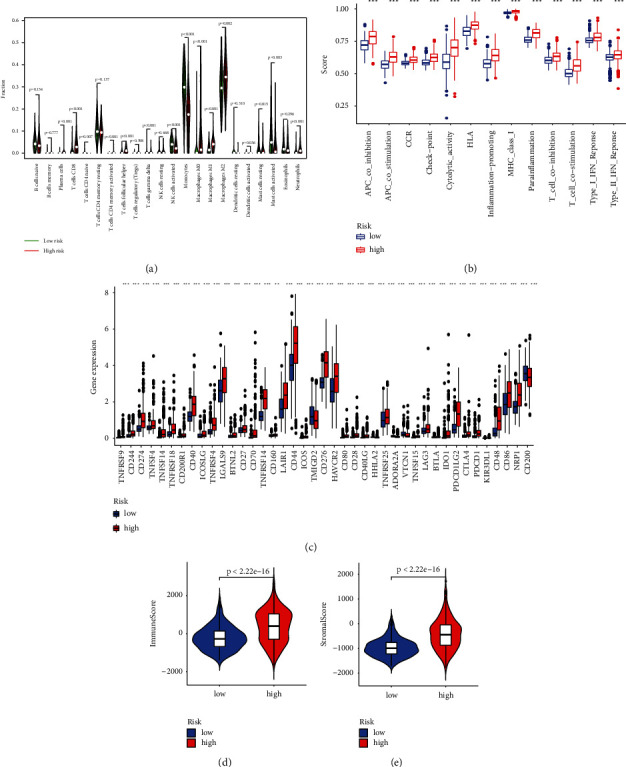
Correlation between prognostic lncRNAs and the immune microenvironment. (a) The immune function between two risk groups. (b) Boxplot of immune scores for two groups. (c) Analysis of immune checkpoints for two differential groups. (d)-(e) Differential analysis of immune and stromal scores.

**Figure 8:**
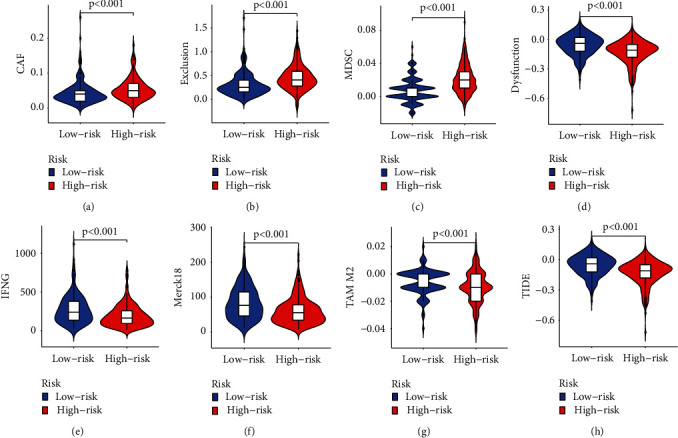
(a) CAF, (b) Exclusion, (c) MDSC, (d)Dysfunction, (e) IFNG, (f) Merck18, (g) TAM M2, and (h) TIDE scores between two risk groups in TCGA.

We further analyzed the activity and enrichment of multiple immune cells, immune pathways, and functions based on the prognostic model. There are significant differences in the expression levels of immune indicators between the low- and high-risk groups. Vioplot indicated more immune cells in the immune microenvironment of the high-risk group, such as CD8+ T cells, monocytes, and macrophages (Figure 7(a)). We next conducted a study of immune function between low-andhigh-risk groups. Immune processes are more aggressive in the high-risk group, e.g., APC coinhibition, APC costimulation, cytolytic activity, and inflammation-promoting (Figure 7(b)). Most immune checkpoints also displayed better activation in the high-risk group. This suggests using appropriate immune checkpoint inhibitors for the high-risk group (Figure 6(c)). Then, the high-risk subgroup in TCGA shows significantly higher scores in immune, stromal, and ESTIMATE scores (Figures 7(d)-7(e)). In addition, the CAF, Exclusion, and MDSC scores were higher in the high-risk subgroup (Figures 8(a)–8(c)), while dysfunction, IFNG, Merck18, TAM M2, and TIDE scores were higher in the low-risk group (Figures 8(d)–8(h)).

### 3.7. Drug Filtering for Necroptosis-Related lncRNA Prognostic Model and Environment Analysis

**Figure 9:**
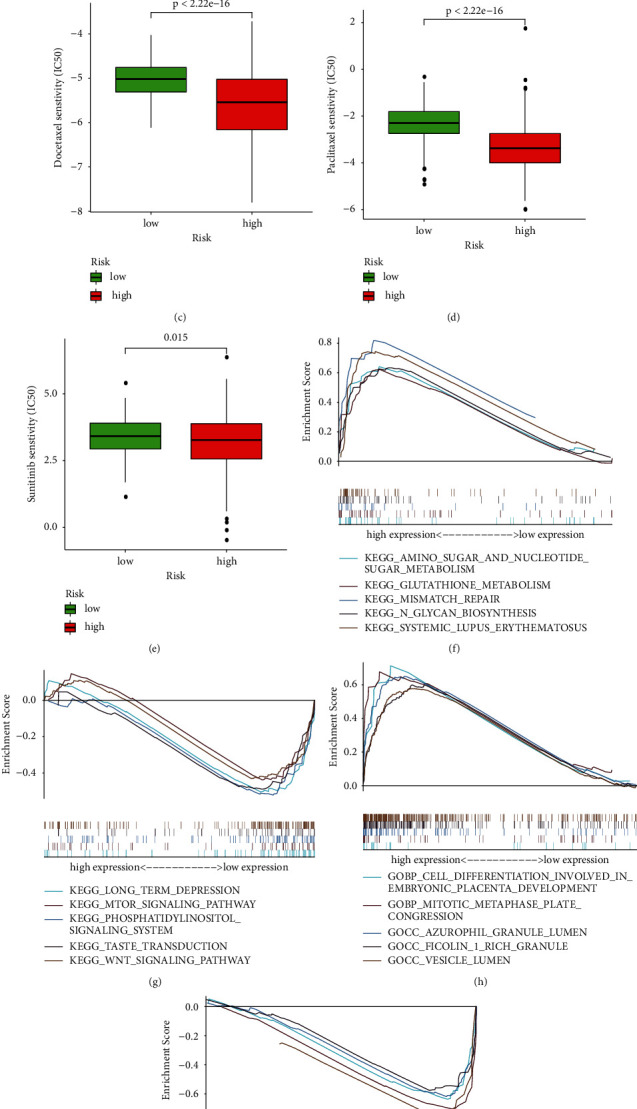
Differential analysis of drug sensitivity and GSEA analysis. (a)–(e) ICI50 of Imatinib, Cisplatin, Docetaxel, Paclitaxel, and Sunitinib between high-risk and low-risk groups.

To investigate potential drug targeting in the prognostic model for glioma patients' treatment, we estimated treatment response by half-maximal inhibitory concentration (IC50). We screened 138 drugs with IC50s that differed significantly between the two groups. The IC50 of Imatinib in the high-risk was higher, while the IC50 of Cisplatin, Docetaxel, Paclitaxel, and Sunitinib was higher in the low-risk group (Figures 9(a)–9(e)). In addition, the top5 KEGG enrichment results of the high-risk and low-risk subgroups were shown in Figures 8(f)–8(g), and GO enrichment results were shown in Figures 9(h)–9(i).

## 4. Discussion

Various cell death modalities in glioma have become a hot topic in the prognostic marker of glioma, where necroptosis, a form of programmed cell death, has demonstrated its robust prognostic ability in gastric, colon, and breast cancers [17, 19]. Identifying a specific and reliable prognostic marker is extremely important to improve the prognosis of glioma patients. While there are many other lncRNA predictive signatures of survival outcomes in glioma patients, necroptosis-related lncRNA predictive signatures have not been reported. We screened prognostic necroptosis-related lncRNA and utilized the prognostic necroptosis-related model to explore comprehensive immune analysis and drug sensitivity. Our study employs a biomarker approach to screen a large number of genes for possible therapeutic targets in glioma. The aim is to provide a new perspective with a limited number of gene markers to provide realistic prognostic assessments and treatment options for patients with glioma. The results of our chosen modeling method are more stable and reproducible, and the predicted prognosis constructed with this method proved to be relatively accurate. In addition, the lncRNAs that were not previously identified in gliomas were screened out, which provides new directions for us to continue our in-depth studies in the future.

Apoptosis resistance exists widely in tumor tissue and is the main obstacle to the success of tumor therapy. Bypassing apoptosis and inducing cancer cell death is an excellent therapeutic strategy. Necroptosis is a novel programmed death that is not regulated by Caspase and is mainly mediated by Receptor-Interacting Protein 1 (RIP1), RIP3, and Mixed Lineage Kinase Domain-Like (MLKL) [20]. RIPK3 causes plasma membrane disruption and cell lysis leading to cell necrosis through phosphorylation of MLKL. Cell-type and environment-based activation of RIPK1 may lead to apoptosis or inflammation [21]. In the mature nervous system, RIPK1 kinase-dependent necroptosis is the primary enforcer of cell death in response to extracellular inflammatory signals.

In this study, we obtained 354 differentially expressed necroptosis-related lncRNAs. 12 necroptosis-related lncRNAs highly associated with OS in glioma patients were identified by lasso and univariate and multifactorial Cox regression, and risk prognostic models were constructed by risk score (i.e., AC025857.2, AC092718.4, AL513534.1, AC083864.2, ZNF236-DT, AC099850.3, AL590094.1, AC010226.1, POLR2J4, AC023024.1, SLC25A21-AS1, and AC109439.2). In these lncRNAs, AC092718.4 has been reported to be highly correlated with ovarian cancer as a predictive signature [22]. AC099850.3 has been found to promote proliferation and invasion in hepatocellular carcinoma via the PRR11/PI3K/AKT pathway and is also a major participant in prognostic models for squamous cell carcinoma of the tongue and non-small-cell lung cancer [23–25]. High expression of AL590094.1 has been found to be a risk factor for patients with clear cell renal cell carcinoma [26]. AC010226.1 as an m6-related lncRNA could be a new therapeutic target for squamous cell carcinoma of the head and neck [27]. POLR2J4 functioned as an oncogene in colorectal through the microRNA-203a-3p.1 and CREB1 axis and is highly expressed in hepatocellular carcinomas [28, 29]. SLC25A21-AS1 as ferroptosis-related lncRNA mediated prognosis associated with immune landscapes and radiotherapy responses in glioma, which may shed some light on our study [30]. AC109439.2 had the potential to be used as an adjunct biomarker for TNM staging and more accurate segmentation of esophageal squamous cell carcinoma patients [31]. Five remaining lncRNAs are reported for the first time (i. e., AC025857.2, AL513534.1, AC083864.2, ZNF236-DT, and AC023024.1). There have been no previous studies on their function. ZNF236-DT is a divergent transcript of its neighboring protein-coding gene ZNF236, located on chromosome 18. AC023024.1 is involved in the degradation process of misfolded proteins in the endoplasmic reticulum and has a role in inflammation control [32]. All 12 lncRNAs can be used as diagnostic and prognostic biomarkers for glioma and function as targets for immunotherapy. Meanwhile, we need further basic experiments to verify their functionality. Our method for screening lncRNAs has been validated, and the model validation approach is common practice with reliable results. The results showed that the low-risk group had a longer OS than the high-risk group and were consistent with clinical reality, indicating that our prognostic model was accurate.

GSEA-GO shows high expression of cranial nerve morphogenesis, deoxyribose phosphate metabolism, anaphylatoxin one rich granules, anaphylatoxin one rich granules lumen, and vesicle lumen, and low expression of retrograde transport endosome to golgi, ubiquitin ligase substrate adaptor activity, torc1 signaling, peptidyl lysine demethylation, and cytoplasmic microtubule organization. GSEA-KEGG indicates high expression of a signaling pathway, that is, systemic lupus erythematosus, *n* glycan biosynthesis, amino sugar, and nucleotide sugar metabolism, cell cycle and glutathione metabolism, and low expression of a signaling pathway, that is, wnt, inositol phosphate metabolism, butanoate metabolism, long-term depression, and taste transduction. These signaling pathways and biological processes may inspire future exploration of glioma formation and treatment mechanisms.

Immunotherapy is currently used in many tumors but is still being explored for gliomas as immune surveillance in the CNS is more complex [33]. At the same time, the CNS has a unique immune microenvironment and has long been considered an immune-privileged site, which has caused some disturbance in the immunotherapy of gliomas. In addition, a study has shown that standard therapies for glioma such as surgery, radiotherapy, temozolomide chemotherapy, and glucocorticoids may all be immunosuppressive, further highlighting the desirability of developing treatment options that target the immune response [34]. Vaccine therapy, oncolytic virus therapy, immune checkpoint inhibitors, and chimeric antigen receptor (CAR) t-cell therapy are the immunotherapeutic modalities currently being investigated in glioma. Current vaccine approaches that may take advantage of the adaptive immune system include rindopepimut, a peptide vaccine against epidermal growth factor receptor (EGFR) variant III [35]. Dendritic cell- (DC-) based vaccines that use autologous tumor tissue to generate tumor antigens have also been developed, such as DCVax-L [36]. A recombinant lysozyme poliovirus PVSRIPO that activates antitumor immune response has improved OS in glioma patients in a trial [37]. Tests primarily targeting PD-1/PD-L1 or CTLA-4 immune checkpoint inhibitors have been conducted in glioma [38]. Recent studies show that GD2-CAR-T cells are effective in treating diffuse midline gliomas with h3k27 m mutations [39]. The efficacy of these immunotherapeutic strategies for glioma has not been fully demonstrated, and their authenticity and efficacy are open to question.

### 4.1. Limitation

We did not perform experimental validation of the prognostic model. The inevitable batch effect also confounded the model to some extent. For a large population of glioma patients, a sample of nearly two thousand is not fully representative of the overall population.

## 5. Conclusion

Predictive models of 12 necroptosis-related lncRNAs can facilitate the assessment of the prognosis and molecular characteristics of glioma patients and improve treatment modalities, which can be further applied in the clinic.

## Figures and Tables

**Table 1:** 159 necroptosis-related genes from KEGG and GSEA.

AIFM1	H2AB1	IFNAR1	SHARPIN
ALOX15	H2AB2	IFNAR2	SLC25A31
BAX	H2AB3	IFNB1	SLC25A4
BCL2	H2AC1	IFNG	SLC25A5
BID	H2AC11	IFNGR1	SLC25A6
BIRC2	H2AC12	IFNGR2	SMPD1
BIRC3	H2AC13	IL1A	SPATA2
CAMK2A	H2AC14	IL1B	SPATA2L
CAMK2B	H2AC15	IL33	SQSTM1
CAMK2D	H2AC16	IRF9	STAT1
CAMK2G	H2AC17	JAK1	STAT2
CAPN1	H2AC18	JAK2	STAT3
CAPN2	H2AC19	JAK3	STAT4
CASP1	H2AC20	JMJD7-PLA2G4B	STAT5A
CASP8	H2AC21	MACROH2A1	STAT5B
CFLAR	H2AC4	MACROH2A2	STAT6
CHMP1A	H2AC6	MAPK10	TICAM1
CHMP1B	H2AC7	MAPK8	TICAM2
CHMP2A	H2AC8	MAPK9	TLR3
CHMP2B	H2AJ	MLKL	TLR4
CHMP3	H2AW	NLRP3	TNF
CHMP4A	H2AX	PARP1	TNFAIP3
CHMP4B	H2AZ1	PGAM5	TNFRSF10A
CHMP4C	H2AZ2	PLA2G4A	TNFRSF10B
CHMP5	HMGB1	PLA2G4B	TNFRSF1A
CHMP6	HSP90AA1	PLA2G4C	TNFSF10
CHMP7	HSP90AB1	PLA2G4D	TRADD
CYBB	IFNA1	PLA2G4E	TRAF2
CYLD	IFNA10	PLA2G4F	TRAF5
DNM1L	IFNA13	PPIA	TRPM7
EIF2AK2	IFNA14	PPID	TYK2
FADD	IFNA16	PYCARD	USP21
FAF1	IFNA17	PYGB	VDAC1
FAS	IFNA2	PYGL	VDAC2
FASLG	IFNA21	PYGM	VDAC3
FTH1	IFNA4	RBCK1	VPS4A
FTL	IFNA5	RIPK1	VPS4B
GLUD1	IFNA6	RIPK3	XIAP
GLUD2	IFNA7	RNF103-CHMP3	ZBP1
GLUL	IFNA8	RNF31	

## Data Availability

The data that support the findings of this study are available at the TCGA (https://tcga-data.nci.nih.gov/tcga/) and GTEx.

## Supplementary Materials

Supplementary Materials Figure S1: association of genes derived from expressional analysis with all lncRNAs with corresponding cor values >0.5. Figure S2: survival analysis of each lncRNA in all glioma patients. Figure S3: among subgroups clustered according to different clinical characteristics, survival differences between high- and low-risk groups. Table S1: prognostic mRNA and their corresponding lncRNA (*P* < 0.001, cor >0.5) after univariate Cox regression analysis.
