# Supplementary material for: Construction and Validation of a Necroptosis-Related lncRNA Signature in Prognosis and Immune Microenvironment for Glioma
Source: J Oncol. 2022 Aug 27;2022:5681206. doi: 10.1155/2022/5681206 (PMC9440826; doi:10.1155/2022/5681206)
Supplement: Supplementary Materials — Figure S1: association of genes derived from expressional analysis with all lncRNAs with corresponding cor values >0.5. Figure S2: survival analysis of each lncRNA in all glioma patients. Figure S3: among subgroups clustered according to different clinical characteristics, survival differences between high- and low-risk groups. Table S1: prognostic mRNA and their corresponding lncRNA (P < 0.001, cor >0.5) after univariate Cox regression analysis. [file 5681206.f1.zip › TableS1.docx]

| gene | lncRNA | cor | pvalue |
| --- | --- | --- | --- |
| FTL | ZNF236-DT | 0.582221 | 1.41E-64 |
| MAPK10 | ZNF236-DT | -0.54018 | 4.01E-54 |
| PPIA | ZNF236-DT | 0.694475 | 1.38E-101 |
| FTL | LINC01503 | 0.613336 | 2.36E-73 |
| TYK2 | AC008735.2 | 0.635905 | 2.33E-80 |
| TLR4 | LINC01338 | 0.5554 | 9.75E-58 |
| TLR4 | AC002451.1 | 0.561038 | 4.00E-59 |
| FTL | AC083799.1 | 0.547476 | 7.82E-56 |
| TYK2 | AL359715.1 | 0.589566 | 1.45E-66 |
| JMJD7-PLA2G4B | AL513534.1 | 0.524774 | 1.19E-50 |
| CYBB | AL034397.3 | 0.506127 | 1.12E-46 |
| IFNGR1 | AL034397.3 | 0.588303 | 3.22E-66 |
| TYK2 | AC073842.2 | 0.504144 | 2.86E-46 |
| JMJD7-PLA2G4B | AC004253.1 | 0.547131 | 9.44E-56 |
| TYK2 | AC004253.1 | 0.561445 | 3.17E-59 |
| JMJD7-PLA2G4B | AL049552.1 | 0.572492 | 5.04E-62 |
| JMJD7-PLA2G4B | AC139887.2 | 0.533454 | 1.38E-52 |
| JMJD7-PLA2G4B | AL391807.1 | 0.688592 | 3.16E-99 |
| PLA2G4B | AL391807.1 | 0.517896 | 3.71E-49 |
| JMJD7-PLA2G4B | AC105206.2 | 0.581554 | 2.12E-64 |
| FTL | BAIAP2-DT | -0.5393 | 6.38E-54 |
| GLUD1 | BAIAP2-DT | 0.547488 | 7.77E-56 |
| PPIA | BAIAP2-DT | -0.57226 | 5.80E-62 |
| CAMK2B | AC107398.3 | 0.513134 | 3.84E-48 |
| MAPK10 | AC107398.3 | 0.673074 | 2.91E-93 |
| CAMK2B | SLC26A4-AS1 | 0.88522 | 1.56E-233 |
| MAPK10 | SLC26A4-AS1 | 0.667814 | 2.53E-91 |
| STAT4 | SLC26A4-AS1 | 0.854656 | 2.62E-200 |
| FTL | AL441992.1 | 0.52778 | 2.58E-51 |
| PPIA | AL441992.1 | 0.544556 | 3.82E-55 |
| JMJD7-PLA2G4B | AC060780.1 | 0.566514 | 1.70E-60 |
| CAMK2B | AC015967.1 | 0.778189 | 9.63E-143 |
| MAPK10 | AC015967.1 | 0.722969 | 7.01E-114 |
| STAT4 | AC015967.1 | 0.752265 | 3.05E-128 |
| JMJD7-PLA2G4B | AC104964.3 | 0.551966 | 6.61E-57 |
| JMJD7-PLA2G4B | AL162586.1 | 0.670686 | 2.23E-92 |
| PLA2G4B | AL162586.1 | 0.501384 | 1.05E-45 |
| JMJD7-PLA2G4B | AP006621.2 | 0.585861 | 1.48E-65 |
| STAT1 | HCP5 | 0.552411 | 5.16E-57 |
| JMJD7-PLA2G4B | AC009120.3 | 0.691823 | 1.62E-100 |
| GLUD1 | AC026401.3 | -0.52213 | 4.50E-50 |
| MAPK10 | AC026401.3 | -0.58462 | 3.20E-65 |
| PPIA | AC026401.3 | 0.581353 | 2.40E-64 |
| FTL | AC093297.2 | -0.57822 | 1.62E-63 |
| JMJD7-PLA2G4B | AP001107.4 | 0.753367 | 8.03E-129 |
| PLA2G4B | AP001107.4 | 0.575553 | 8.10E-63 |
| GLUD1 | AC092718.4 | -0.50324 | 4.38E-46 |
| PPIA | AC092718.4 | 0.555889 | 7.41E-58 |
| TYK2 | AC011462.4 | 0.588377 | 3.07E-66 |
| JMJD7-PLA2G4B | AC008737.1 | 0.547962 | 5.99E-56 |
| TYK2 | AC008737.1 | 0.579872 | 5.93E-64 |
| PPIA | AC064875.1 | 0.565193 | 3.66E-60 |
| CAMK2B | SNAI3-AS1 | 0.679631 | 9.73E-96 |
| MAPK10 | SNAI3-AS1 | 0.555231 | 1.07E-57 |
| STAT4 | SNAI3-AS1 | 0.533284 | 1.51E-52 |
| JMJD7-PLA2G4B | AC087481.3 | 0.735154 | 1.27E-119 |
| PLA2G4B | AC087481.3 | 0.522502 | 3.74E-50 |
| TYK2 | AC087481.3 | 0.515076 | 1.49E-48 |
| JMJD7-PLA2G4B | AC087276.1 | 0.616837 | 2.10E-74 |
| PPIA | AL162231.2 | 0.507098 | 7.04E-47 |
| JMJD7-PLA2G4B | AL355075.2 | 0.70714 | 7.21E-107 |
| CAMK2B | AC004816.1 | 0.774771 | 1.01E-140 |
| MAPK10 | AC004816.1 | 0.601675 | 5.95E-70 |
| STAT4 | AC004816.1 | 0.76141 | 3.81E-133 |
| CYBB | AC093627.7 | 0.602862 | 2.72E-70 |
| IFNGR1 | AC093627.7 | 0.563675 | 8.81E-60 |
| TLR3 | AC093627.7 | 0.541197 | 2.32E-54 |
| FTL | AC009227.1 | -0.53902 | 7.40E-54 |
| GLUD1 | AC009227.1 | 0.537349 | 1.79E-53 |
| PPIA | AC009227.1 | -0.5082 | 4.15E-47 |
| CAMK2B | AC004656.1 | 0.547883 | 6.26E-56 |
| STAT4 | AC004656.1 | 0.599857 | 1.96E-69 |
| JMJD7-PLA2G4B | MUC20-OT1 | 0.571394 | 9.68E-62 |
| CHMP4A | AL136295.7 | 0.542849 | 9.59E-55 |
| GLUD1 | AC124854.1 | 0.516831 | 6.28E-49 |
| GLUD1 | AL109741.1 | 0.579856 | 5.99E-64 |
| FTL | SBF2-AS1 | 0.517146 | 5.38E-49 |
| PPIA | SBF2-AS1 | 0.605258 | 5.55E-71 |
| GLUD1 | TMEM254-AS1 | 0.525851 | 6.90E-51 |
| JMJD7-PLA2G4B | ANKRD10-IT1 | 0.531494 | 3.82E-52 |
| CHMP4A | AC025162.2 | 0.522333 | 4.07E-50 |
| JMJD7-PLA2G4B | CR936218.1 | 0.625887 | 3.53E-77 |
| CAMK2B | AC005330.1 | 0.594624 | 5.82E-68 |
| MAPK10 | AC005330.1 | 0.554592 | 1.53E-57 |
| JMJD7-PLA2G4B | AL512791.1 | 0.563668 | 8.85E-60 |
| CAMK2B | ACAP2-IT1 | 0.638101 | 4.50E-81 |
| MAPK10 | ACAP2-IT1 | 0.632798 | 2.32E-79 |
| STAT4 | ACAP2-IT1 | 0.557236 | 3.47E-58 |
| CYBB | AF127936.1 | 0.621602 | 7.46E-76 |
| IFNGR1 | AF127936.1 | 0.615915 | 3.98E-74 |
| TLR3 | AF127936.1 | 0.508605 | 3.42E-47 |
| FTL | AL353796.1 | -0.501 | 1.26E-45 |
| JMJD7-PLA2G4B | AC009955.4 | 0.574754 | 1.31E-62 |
| PLA2G4B | AC009955.4 | 0.509945 | 1.80E-47 |
| TLR4 | LINC00836 | 0.537203 | 1.94E-53 |
| TYK2 | AC027796.4 | 0.603613 | 1.66E-70 |
| TYK2 | AC005785.1 | 0.545183 | 2.72E-55 |
| CAMK2B | AC015468.3 | 0.773533 | 5.36E-140 |
| MAPK10 | AC015468.3 | 0.578739 | 1.18E-63 |
| STAT4 | AC015468.3 | 0.82026 | 5.12E-171 |
| CHMP4A | AL136295.6 | 0.558297 | 1.90E-58 |
| CAMK2B | LINC00294 | 0.565856 | 2.49E-60 |
| MAPK10 | LINC00294 | 0.628298 | 6.20E-78 |
| TLR3 | LINC00294 | -0.5194 | 1.76E-49 |
| JMJD7-PLA2G4B | AC009118.1 | 0.524935 | 1.10E-50 |
| JMJD7-PLA2G4B | AC110285.1 | 0.544269 | 4.46E-55 |
| JMJD7-PLA2G4B | AP000757.1 | 0.600856 | 1.02E-69 |
| PLA2G4B | AP000757.1 | 0.509053 | 2.76E-47 |
| CAMK2B | AC092376.2 | 0.514871 | 1.64E-48 |
| JMJD7-PLA2G4B | ZNF32-AS1 | 0.550808 | 1.25E-56 |
| STAT4 | CFAP58-DT | 0.522832 | 3.17E-50 |
| JMJD7-PLA2G4B | AC004148.2 | 0.588465 | 2.91E-66 |
| PLA2G4B | AC004148.2 | 0.555065 | 1.18E-57 |
| TYK2 | AC004148.2 | 0.518189 | 3.21E-49 |
| CAMK2B | AC022400.1 | 0.716122 | 8.66E-111 |
| MAPK10 | AC022400.1 | 0.645882 | 1.20E-83 |
| STAT4 | AC022400.1 | 0.706382 | 1.52E-106 |
| GLUD1 | AL391834.2 | 0.54948 | 2.61E-56 |
| MLKL | AC025857.2 | 0.552553 | 4.77E-57 |
| TNFAIP3 | AC025857.2 | 0.536506 | 2.80E-53 |
| JMJD7-PLA2G4B | AC107375.1 | 0.537245 | 1.90E-53 |
| IRF9 | AL356356.1 | 0.511211 | 9.76E-48 |
| JMJD7-PLA2G4B | AC067852.3 | 0.752499 | 2.30E-128 |
| JMJD7-PLA2G4B | AC090589.3 | 0.694799 | 1.02E-101 |
| TYK2 | AC090589.3 | 0.593116 | 1.53E-67 |
| CAMK2B | AC012603.1 | 0.540618 | 3.17E-54 |
| STAT4 | AC012603.1 | 0.569218 | 3.49E-61 |
| JMJD7-PLA2G4B | AC026471.1 | 0.600004 | 1.78E-69 |
| IL1B | AC245128.3 | 0.55548 | 9.32E-58 |
| PPIA | AC099850.3 | 0.535458 | 4.85E-53 |
| CAMK2B | DLGAP1-AS4 | 0.859731 | 2.86E-205 |
| MAPK10 | DLGAP1-AS4 | 0.676555 | 1.44E-94 |
| STAT4 | DLGAP1-AS4 | 0.898933 | 1.13E-251 |
| FTL | BX284668.5 | 0.503855 | 3.28E-46 |
| JMJD7-PLA2G4B | AC008124.1 | 0.529412 | 1.12E-51 |
| CAMK2B | AL365205.3 | 0.68145 | 1.95E-96 |
| MAPK10 | AL365205.3 | 0.523936 | 1.82E-50 |
| STAT4 | AL365205.3 | 0.682965 | 5.07E-97 |
| FTL | AL591848.4 | -0.55543 | 9.58E-58 |
| GLUD1 | AL591848.4 | 0.585873 | 1.47E-65 |
| PPIA | AL591848.4 | -0.53667 | 2.56E-53 |
| CAMK2B | AC138649.1 | 0.790626 | 2.04E-150 |
| MAPK10 | AC138649.1 | 0.596155 | 2.17E-68 |
| STAT4 | AC138649.1 | 0.807788 | 6.55E-162 |
| PPIA | AL355338.1 | 0.528648 | 1.66E-51 |
| CFLAR | AC145098.1 | 0.53165 | 3.53E-52 |
| CYBB | AC145098.1 | 0.692303 | 1.04E-100 |
| FTL | AC145098.1 | 0.530336 | 6.95E-52 |
| MLKL | AC145098.1 | 0.626567 | 2.16E-77 |
| TNFAIP3 | AC145098.1 | 0.531599 | 3.62E-52 |
| FTL | CYTOR | 0.656524 | 2.72E-87 |
| PPIA | CYTOR | 0.693718 | 2.79E-101 |
| GLUD1 | NKAIN3-IT1 | 0.683955 | 2.09E-97 |
| MAPK10 | AL022313.4 | 0.589477 | 1.54E-66 |
| JMJD7-PLA2G4B | AC132872.3 | 0.537843 | 1.38E-53 |
| TYK2 | AC132872.3 | 0.59236 | 2.47E-67 |
| GLUD1 | ZNF710-AS1 | 0.572695 | 4.47E-62 |
| JMJD7-PLA2G4B | AL133325.3 | 0.53948 | 5.80E-54 |
| GLUD1 | FLJ16779 | 0.547488 | 7.77E-56 |
| JMJD7-PLA2G4B | AL031670.1 | 0.686276 | 2.59E-98 |
| TYK2 | AL109811.3 | 0.50087 | 1.33E-45 |
| TYK2 | AC005197.1 | 0.526017 | 6.34E-51 |
| TYK2 | AL139287.1 | 0.539224 | 6.65E-54 |
| JMJD7-PLA2G4B | ERVK13-1 | 0.557568 | 2.88E-58 |
| TYK2 | AL354836.1 | 0.550609 | 1.40E-56 |
| TYK2 | AC021078.1 | 0.501302 | 1.09E-45 |
| FTL | AC112491.1 | 0.537314 | 1.83E-53 |
| PPIA | AC112491.1 | 0.662153 | 2.80E-89 |
| CAMK2B | AL121929.2 | 0.583883 | 5.05E-65 |
| FTL | NORAD | -0.54693 | 1.06E-55 |
| MAPK10 | NORAD | 0.636848 | 1.15E-80 |
| GLUD1 | AC124312.5 | 0.567696 | 8.53E-61 |
| PPIA | AC124312.5 | -0.51609 | 9.05E-49 |
| FTL | AC018730.2 | -0.51254 | 5.11E-48 |
| JMJD7-PLA2G4B | AC018730.2 | 0.567037 | 1.25E-60 |
| FTL | AL157392.3 | -0.6339 | 1.03E-79 |
| GLUD1 | AL157392.3 | 0.579157 | 9.17E-64 |
| JMJD7-PLA2G4B | AL157392.3 | 0.693388 | 3.79E-101 |
| PPIA | AL157392.3 | -0.61543 | 5.58E-74 |
| GLUD1 | AL356056.2 | 0.549607 | 2.43E-56 |
| MAPK10 | AL356056.2 | 0.526177 | 5.84E-51 |
| CAMK2B | AC092171.2 | 0.605082 | 6.24E-71 |
| STAT4 | AC092171.2 | 0.532078 | 2.82E-52 |
| GLUD1 | AC023024.1 | 0.529227 | 1.23E-51 |
| JMJD7-PLA2G4B | SLC9A3-AS1 | 0.657153 | 1.64E-87 |
| PLA2G4B | SLC9A3-AS1 | 0.50467 | 2.23E-46 |
| JMJD7-PLA2G4B | AC009065.5 | 0.560307 | 6.08E-59 |
| JMJD7-PLA2G4B | AC005519.1 | 0.76561 | 1.79E-135 |
| PLA2G4B | AC005519.1 | 0.557538 | 2.93E-58 |
| TYK2 | AC005519.1 | 0.573412 | 2.92E-62 |
| TYK2 | MZF1-AS1 | 0.502581 | 5.98E-46 |
| FTL | LINC00339 | 0.620866 | 1.25E-75 |
| PPIA | LINC00339 | 0.578306 | 1.54E-63 |
| PYGB | LINC00339 | -0.5025 | 6.22E-46 |
| JMJD7-PLA2G4B | CCND2-AS1 | 0.552377 | 5.26E-57 |
| JMJD7-PLA2G4B | AC004982.2 | 0.508121 | 4.32E-47 |
| TYK2 | AC004982.2 | 0.519827 | 1.42E-49 |
| JMJD7-PLA2G4B | AC122129.1 | 0.810436 | 8.69E-164 |
| JMJD7-PLA2G4B | AL132989.1 | 0.741116 | 1.51E-122 |
| CHMP6 | PXN-AS1 | 0.546417 | 1.39E-55 |
| JMJD7-PLA2G4B | AC138028.4 | 0.523072 | 2.81E-50 |
| TYK2 | AC138028.4 | 0.538496 | 9.78E-54 |
| JMJD7-PLA2G4B | ARHGAP27P1-BPTFP1-KPNA2P3 | 0.622676 | 3.49E-76 |
| TYK2 | ARHGAP27P1-BPTFP1-KPNA2P3 | 0.58025 | 4.71E-64 |
| JMJD7-PLA2G4B | GDNF-AS1 | 0.623508 | 1.93E-76 |
| FTL | SLC25A21-AS1 | -0.53218 | 2.68E-52 |
| PPIA | SLC25A21-AS1 | -0.55509 | 1.16E-57 |
| CAMK2B | AL133406.3 | 0.554926 | 1.27E-57 |
| STAT4 | AL133406.3 | 0.589949 | 1.14E-66 |
| CYBB | AC110995.1 | 0.723414 | 4.37E-114 |
| MLKL | AC110995.1 | 0.507448 | 5.96E-47 |
| JMJD7-PLA2G4B | LINC00106 | 0.629047 | 3.60E-78 |
| TYK2 | LINC00106 | 0.539966 | 4.48E-54 |
| GLUD1 | AC009102.2 | 0.673172 | 2.67E-93 |
| GLUD2 | AC009102.2 | 0.532545 | 2.22E-52 |
| JMJD7-PLA2G4B | INE1 | 0.597842 | 7.29E-69 |
| TYK2 | INE1 | 0.526561 | 4.81E-51 |
| GLUD1 | AC048382.5 | 0.505571 | 1.46E-46 |
| FTL | AC005034.5 | -0.51945 | 1.72E-49 |
| CYBB | LINC01150 | 0.739244 | 1.28E-121 |
| FTL | LINC01150 | 0.542279 | 1.30E-54 |
| IFNGR1 | LINC01150 | 0.526677 | 4.53E-51 |
| MLKL | LINC01150 | 0.56511 | 3.84E-60 |
| TLR3 | LINC01150 | 0.630442 | 1.31E-78 |
| CAMK2B | AL139352.1 | 0.541721 | 1.76E-54 |
| MAPK10 | AL139352.1 | 0.53988 | 4.69E-54 |
| STAT4 | AL139352.1 | 0.509228 | 2.54E-47 |
| JMJD7-PLA2G4B | AC021739.5 | 0.593318 | 1.34E-67 |
| TLR4 | LINC01094 | 0.597067 | 1.20E-68 |
| JMJD7-PLA2G4B | LINC00342 | 0.679288 | 1.32E-95 |
| PLA2G4B | LINC00342 | 0.588159 | 3.52E-66 |
| CAMK2B | AC008780.2 | 0.816065 | 7.06E-168 |
| MAPK10 | AC008780.2 | 0.607474 | 1.26E-71 |
| STAT4 | AC008780.2 | 0.835098 | 8.06E-183 |
| CYBB | AC138207.5 | 0.649607 | 6.62E-85 |
| FTL | AC138207.5 | 0.525327 | 8.99E-51 |
| MLKL | AC138207.5 | 0.624854 | 7.39E-77 |
| JMJD7-PLA2G4B | AC114730.3 | 0.708203 | 2.52E-107 |
| PLA2G4B | AC114730.3 | 0.550179 | 1.78E-56 |
| TYK2 | AC114730.3 | 0.541967 | 1.54E-54 |
| JMJD7-PLA2G4B | AL683813.1 | 0.614238 | 1.27E-73 |
| TYK2 | LENG8-AS1 | 0.538192 | 1.15E-53 |
| CAMK2B | RFPL1S | 0.84282 | 1.94E-189 |
| MAPK10 | RFPL1S | 0.732363 | 2.80E-118 |
| STAT4 | RFPL1S | 0.835578 | 3.20E-183 |
| JMJD7-PLA2G4B | Z83843.1 | 0.681233 | 2.36E-96 |
| FTL | AC007619.1 | -0.51639 | 7.80E-49 |
| GLUD1 | AC007619.1 | 0.524867 | 1.14E-50 |
| MAPK10 | AC007619.1 | 0.51682 | 6.32E-49 |
| CYBB | AC002091.1 | 0.669298 | 7.24E-92 |
| IFNGR1 | AC002091.1 | 0.604399 | 9.83E-71 |
| MLKL | AC002091.1 | 0.572932 | 3.88E-62 |
| TLR3 | AC002091.1 | 0.577996 | 1.85E-63 |
| JMJD7-PLA2G4B | AC004492.1 | 0.520872 | 8.46E-50 |
| TYK2 | AC010326.3 | 0.528085 | 2.21E-51 |
| MAPK10 | LBX2-AS1 | -0.55463 | 1.50E-57 |
| JMJD7-PLA2G4B | AP003392.1 | 0.597518 | 8.99E-69 |
| CAMK2B | MIR7-3HG | 0.637291 | 8.27E-81 |
| GLUD1 | AL122035.1 | 0.514461 | 2.01E-48 |
| FTL | MIR9-3HG | -0.50904 | 2.77E-47 |
| JMJD7-PLA2G4B | MIR9-3HG | 0.63252 | 2.85E-79 |
| JMJD7-PLA2G4B | AC013403.2 | 0.534727 | 7.11E-53 |
| GLUD1 | AL450326.1 | 0.511339 | 9.17E-48 |
| MAPK10 | AL450326.1 | 0.52612 | 6.01E-51 |
| FTL | AC073611.1 | 0.635702 | 2.71E-80 |
| PPIA | AC073611.1 | 0.607898 | 9.47E-72 |
| FTL | AL133415.1 | 0.617145 | 1.70E-74 |
| MAPK10 | AL133415.1 | -0.50217 | 7.23E-46 |
| PPIA | AL133415.1 | 0.744265 | 3.98E-124 |
| TYK2 | AC008969.1 | 0.535583 | 4.54E-53 |
| FTL | AL158212.3 | -0.61739 | 1.43E-74 |
| GLUD1 | AL158212.3 | 0.601601 | 6.25E-70 |
| MAPK10 | AL158212.3 | 0.744871 | 1.97E-124 |
| PPIA | AL158212.3 | -0.61352 | 2.07E-73 |
| JMJD7-PLA2G4B | AC008764.2 | 0.535427 | 4.93E-53 |
| TLR4 | AC073941.1 | 0.617424 | 1.40E-74 |
| TYK2 | AC022150.2 | 0.534786 | 6.89E-53 |
| CHMP6 | AC133552.5 | 0.521243 | 7.03E-50 |
| CAMK2B | ATP2B1-AS1 | 0.784646 | 1.16E-146 |
| MAPK10 | ATP2B1-AS1 | 0.542279 | 1.30E-54 |
| STAT4 | ATP2B1-AS1 | 0.739694 | 7.66E-122 |
| CAMK2B | PRKAG2-AS1 | 0.705379 | 4.06E-106 |
| STAT4 | PRKAG2-AS1 | 0.706428 | 1.45E-106 |
| JMJD7-PLA2G4B | AL360181.2 | 0.702617 | 5.97E-105 |
| FTL | AC018797.2 | 0.532129 | 2.75E-52 |
| PPIA | AC018797.2 | 0.538041 | 1.24E-53 |
| TYK2 | TMEM147-AS1 | 0.584597 | 3.25E-65 |
| CAMK2B | AC092720.2 | 0.725362 | 5.52E-115 |
| MAPK10 | AC092720.2 | 0.65611 | 3.80E-87 |
| STAT4 | AC092720.2 | 0.637816 | 5.58E-81 |
| JMJD7-PLA2G4B | AF111169.3 | 0.542446 | 1.19E-54 |
| PPIA | LINC02035 | -0.52633 | 5.41E-51 |
| CAMK2B | AC148477.1 | 0.544714 | 3.51E-55 |
| MAPK10 | AC148477.1 | 0.529447 | 1.10E-51 |
| STAT4 | AC148477.1 | 0.522343 | 4.05E-50 |
| JMJD7-PLA2G4B | AL928654.2 | 0.656767 | 2.24E-87 |
| TYK2 | AL928654.2 | 0.617851 | 1.04E-74 |
| JMJD7-PLA2G4B | AL022328.1 | 0.723856 | 2.74E-114 |
| PLA2G4B | AL022328.1 | 0.543905 | 5.43E-55 |
| FTL | PVT1 | 0.587249 | 6.23E-66 |
| GLUD1 | PVT1 | -0.56485 | 4.47E-60 |
| MAPK10 | PVT1 | -0.54668 | 1.21E-55 |
| PPIA | PVT1 | 0.680382 | 5.02E-96 |
| TYK2 | LINC00265 | 0.591618 | 3.97E-67 |
| GLUD1 | AC087241.1 | 0.532943 | 1.80E-52 |
| CAMK2B | AC021683.1 | 0.689293 | 1.66E-99 |
| MAPK10 | AC021683.1 | 0.551086 | 1.08E-56 |
| STAT4 | AC021683.1 | 0.632437 | 3.03E-79 |
| TYK2 | AC131009.3 | 0.519499 | 1.68E-49 |
| IL1B | AC243960.3 | 0.541456 | 2.02E-54 |
| TNF | AC243960.3 | 0.54926 | 2.94E-56 |
| CAMK2B | AC124312.2 | 0.523114 | 2.75E-50 |
| MAPK10 | AC124312.2 | 0.745337 | 1.14E-124 |
| FTL | PAXBP1-AS1 | -0.5152 | 1.40E-48 |
| JMJD7-PLA2G4B | PAXBP1-AS1 | 0.626772 | 1.87E-77 |
| TYK2 | AL359715.2 | 0.504313 | 2.64E-46 |
| TYK2 | AL031186.1 | 0.516528 | 7.29E-49 |
| CYBB | AL161785.1 | 0.723269 | 5.10E-114 |
| IFNGR1 | AL161785.1 | 0.564162 | 6.65E-60 |
| MLKL | AL161785.1 | 0.50363 | 3.65E-46 |
| TLR3 | AL161785.1 | 0.517998 | 3.53E-49 |
| CAMK2B | AC005726.3 | 0.593247 | 1.40E-67 |
| STAT4 | AC005726.3 | 0.640979 | 5.13E-82 |
| JMJD7-PLA2G4B | AC087741.1 | 0.525672 | 7.55E-51 |
| CAMK2B | HAR1A | 0.617629 | 1.21E-74 |
| MAPK10 | HAR1A | 0.614831 | 8.42E-74 |
| STAT4 | HAR1A | 0.60029 | 1.48E-69 |
| CAMK2B | AC125616.1 | 0.792364 | 1.57E-151 |
| MAPK10 | AC125616.1 | 0.665622 | 1.58E-90 |
| STAT4 | AC125616.1 | 0.849775 | 1.03E-195 |
| JMJD7-PLA2G4B | AC022098.1 | 0.541073 | 2.48E-54 |
| TYK2 | AC022098.1 | 0.595426 | 3.47E-68 |
| JMJD7-PLA2G4B | AC245060.2 | 0.582281 | 1.36E-64 |
| JMJD7-PLA2G4B | U73166.1 | 0.522815 | 3.20E-50 |
| CHMP4A | AC135178.5 | 0.535581 | 4.55E-53 |
| CAMK2B | LINC00957 | 0.701951 | 1.14E-104 |
| STAT4 | LINC00957 | 0.665403 | 1.90E-90 |
| CAMK2B | AL021395.1 | 0.870957 | 5.67E-217 |
| MAPK10 | AL021395.1 | 0.709868 | 4.82E-108 |
| STAT4 | AL021395.1 | 0.873298 | 1.50E-219 |
| JMJD7-PLA2G4B | SLC25A25-AS1 | 0.566176 | 2.07E-60 |
| TYK2 | SLC25A25-AS1 | 0.506973 | 7.47E-47 |
| FTL | AC020915.3 | 0.541109 | 2.44E-54 |
| GLUD1 | AC020915.3 | -0.54258 | 1.11E-54 |
| MAPK10 | AC020915.3 | -0.52874 | 1.58E-51 |
| PPIA | AC020915.3 | 0.647193 | 4.35E-84 |
| FTL | SNHG14 | -0.55617 | 6.32E-58 |
| GLUD1 | SNHG14 | 0.500651 | 1.48E-45 |
| JMJD7-PLA2G4B | SNHG14 | 0.580541 | 3.94E-64 |
| PPIA | SNHG14 | -0.51747 | 4.59E-49 |
| JMJD7-PLA2G4B | LINCR-0001 | 0.666112 | 1.05E-90 |
| JMJD7-PLA2G4B | SLC6A1-AS1 | 0.576692 | 4.08E-63 |
| FTL | AC147651.3 | 0.682354 | 8.73E-97 |
| MLKL | AC147651.3 | 0.520435 | 1.05E-49 |
| PPIA | AC147651.3 | 0.584908 | 2.68E-65 |
| CYBB | AC008760.2 | 0.605613 | 4.38E-71 |
| CAMK2B | MIR600HG | 0.716878 | 3.99E-111 |
| MAPK10 | MIR600HG | 0.672987 | 3.13E-93 |
| STAT4 | MIR600HG | 0.722731 | 9.00E-114 |
| FTL | CARD8-AS1 | 0.720635 | 8.12E-113 |
| PPIA | CARD8-AS1 | 0.548907 | 3.57E-56 |
| FTL | ZNF529-AS1 | 0.515844 | 1.02E-48 |
| MAPK10 | ZNF529-AS1 | -0.50001 | 1.99E-45 |
| PPIA | ZNF529-AS1 | 0.549621 | 2.41E-56 |
| TYK2 | DM1-AS | 0.561535 | 3.01E-59 |
| JMJD7-PLA2G4B | RAD51-AS1 | 0.729342 | 7.62E-117 |
| PLA2G4B | RAD51-AS1 | 0.550615 | 1.40E-56 |
| TYK2 | RAD51-AS1 | 0.504503 | 2.41E-46 |
| JMJD7-PLA2G4B | AC103974.1 | 0.534422 | 8.34E-53 |
| FTL | AL117332.1 | 0.536255 | 3.19E-53 |
| GLUD1 | AL117332.1 | -0.57355 | 2.68E-62 |
| MAPK10 | AL117332.1 | -0.54831 | 4.96E-56 |
| PPIA | AL117332.1 | 0.618484 | 6.67E-75 |
| GLUD1 | AC109439.2 | 0.532052 | 2.86E-52 |
| CYBB | AL133371.2 | 0.709171 | 9.64E-108 |
| IFNGR1 | AL133371.2 | 0.538044 | 1.24E-53 |
| MLKL | AL133371.2 | 0.5397 | 5.16E-54 |
| TLR3 | AL133371.2 | 0.515048 | 1.51E-48 |
| JMJD7-PLA2G4B | AC084018.1 | 0.66975 | 4.94E-92 |
| PLA2G4B | AC084018.1 | 0.522307 | 4.13E-50 |
| JMJD7-PLA2G4B | AC007666.1 | 0.527191 | 3.49E-51 |
| GLUD1 | WDFY3-AS2 | 0.648024 | 2.28E-84 |
| MAPK10 | WDFY3-AS2 | 0.50996 | 1.79E-47 |
| PPIA | WDFY3-AS2 | -0.50399 | 3.07E-46 |
| GLUD1 | AL049838.1 | 0.513905 | 2.64E-48 |
| JMJD7-PLA2G4B | AL450263.1 | 0.658267 | 6.67E-88 |
| CAMK2B | AP003355.2 | 0.878884 | 6.43E-226 |
| MAPK10 | AP003355.2 | 0.679184 | 1.44E-95 |
| STAT4 | AP003355.2 | 0.887635 | 1.48E-236 |
| JMJD7-PLA2G4B | AC245060.5 | 0.591176 | 5.25E-67 |
| PLA2G4B | AC245060.5 | 0.510616 | 1.30E-47 |
| CYBB | AC138207.4 | 0.596037 | 2.34E-68 |
| MLKL | AC138207.4 | 0.592148 | 2.83E-67 |
| TYK2 | AC015813.1 | 0.589188 | 1.84E-66 |
| JMJD7-PLA2G4B | AC011477.2 | 0.511859 | 7.13E-48 |
| JMJD7-PLA2G4B | AC015849.3 | 0.606394 | 2.60E-71 |
| TYK2 | AC015849.3 | 0.515346 | 1.30E-48 |
| JMJD7-PLA2G4B | AL033384.2 | 0.549874 | 2.10E-56 |
| GLUD1 | AC124312.4 | 0.553776 | 2.42E-57 |
| MAPK10 | AC124312.4 | 0.519242 | 1.91E-49 |
| HSP90AB1 | LLPH-DT | 0.644803 | 2.76E-83 |
| MAPK10 | AC027307.2 | -0.59277 | 1.91E-67 |
| IRF9 | PTOV1-AS2 | 0.518138 | 3.29E-49 |
| TYK2 | PTOV1-AS2 | 0.707036 | 7.99E-107 |
| FTL | AC120036.4 | -0.63004 | 1.75E-78 |
| GLUD1 | AC120036.4 | 0.561876 | 2.48E-59 |
| MAPK10 | AC120036.4 | 0.686756 | 1.68E-98 |
| MLKL | AC120036.4 | -0.50488 | 2.02E-46 |
| PLA2G4A | AC120036.4 | -0.50302 | 4.86E-46 |
| PPIA | AC120036.4 | -0.56029 | 6.13E-59 |
| JMJD7-PLA2G4B | AC024075.3 | 0.515704 | 1.09E-48 |
| JMJD7-PLA2G4B | AC006435.2 | 0.727116 | 8.44E-116 |
| PLA2G4B | AC006435.2 | 0.55417 | 1.94E-57 |
| TYK2 | AC006435.2 | 0.609046 | 4.37E-72 |
| FTL | AP003486.1 | -0.61304 | 2.89E-73 |
| GLUD1 | AP003486.1 | 0.565972 | 2.33E-60 |
| MAPK10 | AP003486.1 | 0.527588 | 2.85E-51 |
| PPIA | AP003486.1 | -0.55826 | 1.95E-58 |
| CAMK2B | AL365361.1 | 0.656893 | 2.02E-87 |
| MAPK10 | AL365361.1 | 0.54953 | 2.54E-56 |
| STAT4 | AL365361.1 | 0.692613 | 7.80E-101 |
| JMJD7-PLA2G4B | AC020558.2 | 0.680739 | 3.66E-96 |
| PLA2G4B | AC020558.2 | 0.545142 | 2.78E-55 |
| TYK2 | AC020558.2 | 0.53521 | 5.52E-53 |
| GLUD1 | RNF219-AS1 | 0.649042 | 1.03E-84 |
| TLR4 | RNF219-AS1 | 0.552975 | 3.78E-57 |
| JMJD7-PLA2G4B | AC004908.1 | 0.631482 | 6.10E-79 |
| TYK2 | AC245884.8 | 0.503087 | 4.71E-46 |
| CFLAR | NEAT1 | 0.523864 | 1.88E-50 |
| FTL | PSMB8-AS1 | 0.514843 | 1.67E-48 |
| MLKL | PSMB8-AS1 | 0.52119 | 7.22E-50 |
| STAT1 | PSMB8-AS1 | 0.511864 | 7.11E-48 |
| TLR3 | PSMB8-AS1 | 0.565882 | 2.45E-60 |
| CAMK2B | LINC00507 | 0.775928 | 2.11E-141 |
| MAPK10 | LINC00507 | 0.586842 | 8.04E-66 |
| STAT4 | LINC00507 | 0.829183 | 5.59E-178 |
| CAMK2B | DPP10-AS1 | 0.516243 | 8.39E-49 |
| STAT4 | DPP10-AS1 | 0.559453 | 9.89E-59 |
| CAMK2B | PWAR6 | 0.65809 | 7.70E-88 |
| FTL | PWAR6 | -0.53052 | 6.34E-52 |
| GLUD1 | PWAR6 | 0.519214 | 1.93E-49 |
| MAPK10 | PWAR6 | 0.807496 | 1.05E-161 |
| STAT4 | PWAR6 | 0.625821 | 3.70E-77 |
| CYBB | AC090559.1 | 0.844265 | 1.02E-190 |
| IFNGR1 | AC090559.1 | 0.611022 | 1.14E-72 |
| TLR3 | AC090559.1 | 0.621365 | 8.82E-76 |
| CAMK2B | AC015726.1 | 0.563445 | 1.01E-59 |
| STAT4 | AC015726.1 | 0.555967 | 7.09E-58 |
| CHMP4A | AC073896.4 | 0.583956 | 4.83E-65 |
| CHMP6 | AC073896.4 | 0.501637 | 9.31E-46 |
| JMJD7-PLA2G4B | MIR3936HG | 0.505566 | 1.46E-46 |
| CHMP6 | MAFG-DT | 0.534657 | 7.38E-53 |
| JMJD7-PLA2G4B | AL049840.1 | 0.678793 | 2.03E-95 |
| TYK2 | STAG3L5P-PVRIG2P-PILRB | 0.587923 | 4.08E-66 |
| CAMK2B | PART1 | 0.845729 | 5.03E-192 |
| MAPK10 | PART1 | 0.670341 | 2.99E-92 |
| STAT4 | PART1 | 0.893458 | 3.91E-244 |
| GLUD1 | AC021739.3 | 0.507242 | 6.57E-47 |
| JMJD7-PLA2G4B | AC021739.3 | 0.609484 | 3.25E-72 |
| JMJD7-PLA2G4B | ZNF32-AS2 | 0.750188 | 3.70E-127 |
| PLA2G4B | ZNF32-AS2 | 0.531714 | 3.41E-52 |
| JMJD7-PLA2G4B | TRAPPC12-AS1 | 0.520753 | 8.98E-50 |
| PPIA | AC010226.1 | 0.545462 | 2.34E-55 |
| TYK2 | CIRBP-AS1 | 0.542778 | 9.96E-55 |
| FTL | MIR4435-2HG | 0.643414 | 8.02E-83 |
| GLUD1 | MIR4435-2HG | -0.51384 | 2.73E-48 |
| PPIA | MIR4435-2HG | 0.708543 | 1.80E-107 |
| FTL | AC010834.3 | -0.51031 | 1.51E-47 |
| JMJD7-PLA2G4B | AC010834.3 | 0.644343 | 3.93E-83 |
| TLR3 | PAXIP1-AS2 | 0.510404 | 1.44E-47 |
| JMJD7-PLA2G4B | AL391121.1 | 0.501059 | 1.22E-45 |
| JMJD7-PLA2G4B | LINC00599 | 0.542143 | 1.40E-54 |
| FTL | LINC00641 | -0.57817 | 1.67E-63 |
| GLUD1 | LINC00641 | 0.63229 | 3.37E-79 |
| MAPK10 | LINC00641 | 0.801322 | 1.90E-157 |
| PPIA | LINC00641 | -0.56557 | 2.93E-60 |
| JMJD7-PLA2G4B | AC093110.1 | 0.614109 | 1.39E-73 |
| CAMK2B | EMX2OS | 0.616038 | 3.66E-74 |
| MAPK10 | EMX2OS | 0.560456 | 5.58E-59 |
| STAT4 | EMX2OS | 0.603418 | 1.88E-70 |
| CAMK2B | AL356479.1 | 0.733861 | 5.36E-119 |
| MAPK10 | AL356479.1 | 0.54606 | 1.69E-55 |
| STAT4 | AL356479.1 | 0.624665 | 8.46E-77 |
| FTL | WAC-AS1 | -0.52458 | 1.31E-50 |
| PPIA | WAC-AS1 | -0.52531 | 9.05E-51 |
| JMJD7-PLA2G4B | LINC01786 | 0.752268 | 3.04E-128 |
| PLA2G4B | LINC01786 | 0.536492 | 2.82E-53 |
| TYK2 | LINC01786 | 0.515783 | 1.05E-48 |
| CAMK2B | AC061961.1 | 0.787535 | 1.84E-148 |
| MAPK10 | AC061961.1 | 0.714361 | 5.22E-110 |
| STAT4 | AC061961.1 | 0.772663 | 1.72E-139 |
| JMJD7-PLA2G4B | AC053513.1 | 0.659605 | 2.25E-88 |
| FTL | OTUD6B-AS1 | -0.50127 | 1.11E-45 |
| GLUD1 | OTUD6B-AS1 | 0.53588 | 3.89E-53 |
| MAPK10 | OTUD6B-AS1 | 0.648367 | 1.74E-84 |
| PPIA | OTUD6B-AS1 | -0.50469 | 2.21E-46 |
| JMJD7-PLA2G4B | AC009041.2 | 0.540062 | 4.26E-54 |
| FTL | AC254562.3 | -0.53927 | 6.50E-54 |
| JMJD7-PLA2G4B | AC254562.3 | 0.584986 | 2.55E-65 |
| CAMK2B | AL450992.2 | 0.618896 | 5.00E-75 |
| STAT4 | AL450992.2 | 0.550067 | 1.89E-56 |
| GLUD1 | AC004067.1 | -0.55503 | 1.20E-57 |
| PPIA | AC004067.1 | 0.525948 | 6.56E-51 |
| JMJD7-PLA2G4B | AL121772.3 | 0.559104 | 1.21E-58 |
| FTL | LINC00324 | 0.593771 | 1.01E-67 |
| JMJD7-PLA2G4B | TTC28-AS1 | 0.604378 | 9.96E-71 |
| PLA2G4B | TTC28-AS1 | 0.524546 | 1.34E-50 |
| JMJD7-PLA2G4B | AC092119.2 | 0.557302 | 3.34E-58 |
| PLA2G4B | AC092119.2 | 0.50435 | 2.60E-46 |
| MAPK10 | AL662844.4 | 0.527067 | 3.71E-51 |
| JMJD7-PLA2G4B | AL590666.2 | 0.741152 | 1.45E-122 |
| PLA2G4B | AL590666.2 | 0.528757 | 1.57E-51 |
| CYBB | AC087273.2 | 0.502893 | 5.16E-46 |
| JMJD7-PLA2G4B | AC009120.2 | 0.608691 | 5.55E-72 |
| PLA2G4B | AC009120.2 | 0.504426 | 2.50E-46 |
| TYK2 | AC009120.2 | 0.551865 | 6.99E-57 |
| JMJD7-PLA2G4B | AC135050.5 | 0.567581 | 9.12E-61 |
| TYK2 | AC116914.2 | 0.578848 | 1.11E-63 |
| CYBB | PCED1B-AS1 | 0.678186 | 3.46E-95 |
| FTL | PCED1B-AS1 | 0.665326 | 2.03E-90 |
| MLKL | PCED1B-AS1 | 0.619507 | 3.26E-75 |
| TLR3 | PCED1B-AS1 | 0.517996 | 3.53E-49 |
| FTL | AC008669.1 | -0.53006 | 8.03E-52 |
| PPIA | AC008669.1 | -0.50536 | 1.61E-46 |
| JMJD7-PLA2G4B | AC110285.2 | 0.506678 | 8.60E-47 |
| TYK2 | AL365330.1 | 0.573291 | 3.14E-62 |
| HSP90AB1 | POLH-AS1 | 0.666024 | 1.13E-90 |
| PPIA | AC009403.1 | 0.552273 | 5.58E-57 |
| FTL | AL109811.2 | 0.519936 | 1.35E-49 |
| PPIA | AL109811.2 | 0.517754 | 3.98E-49 |
| IFNGR1 | AC012150.2 | 0.513209 | 3.70E-48 |
| JMJD7-PLA2G4B | LINC00928 | 0.532245 | 2.59E-52 |
| JMJD7-PLA2G4B | AL031709.1 | 0.61577 | 4.40E-74 |
| TYK2 | LINC00926 | 0.627852 | 8.56E-78 |
| GLUD1 | AC083864.2 | 0.507451 | 5.95E-47 |
| JMJD7-PLA2G4B | AC129510.1 | 0.562137 | 2.13E-59 |
| TYK2 | AC129510.1 | 0.543789 | 5.78E-55 |
| JMJD7-PLA2G4B | AC127024.4 | 0.549824 | 2.16E-56 |
| JMJD7-PLA2G4B | LINC00632 | 0.500981 | 1.27E-45 |
| PPIA | AC091729.3 | 0.511253 | 9.56E-48 |
| PPIA | LINC00665 | 0.655031 | 9.03E-87 |
| JMJD7-PLA2G4B | GABPB1-AS1 | 0.718826 | 5.34E-112 |
| PLA2G4B | GABPB1-AS1 | 0.552759 | 4.26E-57 |
| FTL | AL590094.1 | 0.524351 | 1.47E-50 |
| PPIA | AL590094.1 | 0.529078 | 1.33E-51 |
| MAPK10 | AC023301.1 | 0.538433 | 1.01E-53 |
| JMJD7-PLA2G4B | AL133367.1 | 0.546157 | 1.60E-55 |
| JMJD7-PLA2G4B | NCAM1-AS1 | 0.706139 | 1.93E-106 |
| TYK2 | ASMTL-AS1 | 0.52302 | 2.88E-50 |
| TLR4 | WDR11-AS1 | 0.504421 | 2.51E-46 |
| CYBB | AC098613.1 | 0.685773 | 4.08E-98 |
| FTL | AC098613.1 | 0.527605 | 2.82E-51 |
| MLKL | AC098613.1 | 0.531664 | 3.50E-52 |
| TLR3 | AC098613.1 | 0.591897 | 3.32E-67 |
| JMJD7-PLA2G4B | RUSC1-AS1 | 0.565379 | 3.29E-60 |
| JMJD7-PLA2G4B | AL022328.2 | 0.723092 | 6.15E-114 |
| PLA2G4B | AL022328.2 | 0.604235 | 1.10E-70 |
| TYK2 | AL022328.2 | 0.563271 | 1.11E-59 |
| JMJD7-PLA2G4B | AC108010.1 | 0.521646 | 5.75E-50 |
| JMJD7-PLA2G4B | AC084125.2 | 0.713164 | 1.76E-109 |
| PLA2G4B | AC084125.2 | 0.508608 | 3.42E-47 |
| TYK2 | AC020907.4 | 0.563416 | 1.02E-59 |
| JMJD7-PLA2G4B | AL031600.1 | 0.768575 | 3.82E-137 |
| PLA2G4B | AL031600.1 | 0.554916 | 1.28E-57 |
| TYK2 | AL031600.1 | 0.508643 | 3.36E-47 |
| JMJD7-PLA2G4B | LINC02449 | 0.585227 | 2.20E-65 |
| JMJD7-PLA2G4B | KCNIP2-AS1 | 0.538637 | 9.08E-54 |
| GLUD1 | POLR2J4 | -0.50298 | 4.97E-46 |
| PPIA | POLR2J4 | 0.68382 | 2.36E-97 |
| JMJD7-PLA2G4B | AC005962.1 | 0.510843 | 1.17E-47 |
| GLUD1 | AC073486.1 | 0.510271 | 1.54E-47 |
| TLR4 | AC073486.1 | 0.501646 | 9.27E-46 |
| CAMK2B | OIP5-AS1 | 0.688079 | 5.04E-99 |
| MAPK10 | OIP5-AS1 | 0.729596 | 5.78E-117 |
| STAT4 | OIP5-AS1 | 0.728742 | 1.46E-116 |
| FTL | MIR155HG | 0.579554 | 7.20E-64 |
| PPIA | MIR155HG | 0.577673 | 2.26E-63 |
| TYK2 | AC069281.2 | 0.54795 | 6.03E-56 |
| JMJD7-PLA2G4B | AL035563.1 | 0.506089 | 1.14E-46 |
| TYK2 | AC005261.1 | 0.511138 | 1.01E-47 |
| FTL | AL445524.1 | 0.701319 | 2.09E-104 |
| GLUD1 | AL445524.1 | -0.55025 | 1.71E-56 |
| MAPK10 | AL445524.1 | -0.54157 | 1.90E-54 |
| PPIA | AL445524.1 | 0.641969 | 2.42E-82 |
| JMJD7-PLA2G4B | AC007663.4 | 0.572228 | 5.90E-62 |
| TYK2 | PDXDC2P-NPIPB14P | 0.535797 | 4.06E-53 |
| JMJD7-PLA2G4B | AL354733.3 | 0.638886 | 2.50E-81 |
| JMJD7-PLA2G4B | VPS9D1-AS1 | 0.581188 | 2.65E-64 |
| TYK2 | VPS9D1-AS1 | 0.524579 | 1.31E-50 |
| JMJD7-PLA2G4B | AC037459.3 | 0.652467 | 6.95E-86 |
| FTL | AP001486.2 | -0.59038 | 8.70E-67 |
| GLUD1 | AP001486.2 | 0.642777 | 1.31E-82 |
| MAPK10 | AP001486.2 | 0.712343 | 4.03E-109 |
| PPIA | AP001486.2 | -0.59005 | 1.07E-66 |
| MAPK10 | AC005696.4 | 0.513653 | 2.98E-48 |
| PLA2G4A | AC005696.4 | -0.51238 | 5.55E-48 |
| PYGB | AC005696.4 | 0.511188 | 9.87E-48 |
| CAMK2B | AC010980.2 | 0.586185 | 1.21E-65 |
| STAT4 | AC010980.2 | 0.518697 | 2.50E-49 |
| JMJD7-PLA2G4B | AC009065.2 | 0.587967 | 3.97E-66 |
| TYK2 | AC009065.2 | 0.538035 | 1.25E-53 |
| CAMK2B | AF106564.1 | 0.665386 | 1.93E-90 |
| MAPK10 | AF106564.1 | 0.599429 | 2.59E-69 |
| STAT4 | AF106564.1 | 0.677993 | 4.10E-95 |
| FTL | LINC00900 | 0.50939 | 2.35E-47 |
| PPIA | LINC00900 | 0.507171 | 6.80E-47 |
| IFNGR1 | LINC01736 | 0.548737 | 3.92E-56 |
| TLR3 | LINC01736 | 0.574688 | 1.36E-62 |
| CAMK2B | AC104836.1 | 0.537457 | 1.69E-53 |
| MAPK10 | AC104836.1 | 0.513169 | 3.77E-48 |
| JMJD7-PLA2G4B | AC066613.1 | 0.647553 | 3.29E-84 |
| CAMK2B | AC110491.1 | 0.782132 | 4.03E-145 |
| MAPK10 | AC110491.1 | 0.640559 | 7.06E-82 |
| STAT4 | AC110491.1 | 0.773455 | 5.96E-140 |
| MAPK10 | MIR124-2HG | 0.557628 | 2.78E-58 |
| CAMK2B | AC010624.2 | 0.540514 | 3.35E-54 |
| TLR4 | LINC01088 | 0.558027 | 2.22E-58 |
| JMJD7-PLA2G4B | ZKSCAN2-DT | 0.563494 | 9.78E-60 |
| PLA2G4B | ZKSCAN2-DT | 0.503961 | 3.12E-46 |
| FTL | AC104825.1 | -0.5186 | 2.62E-49 |
| CAMK2B | AC104072.1 | 0.849421 | 2.20E-195 |
| MAPK10 | AC104072.1 | 0.576013 | 6.14E-63 |
| STAT4 | AC104072.1 | 0.742147 | 4.62E-123 |
| TNFAIP3 | MIR22HG | 0.509947 | 1.80E-47 |
| PPIA | AL355974.2 | 0.542961 | 9.03E-55 |
| PPIA | AC010319.4 | 0.533686 | 1.22E-52 |
| FTL | AC093673.1 | 0.586607 | 9.30E-66 |
| GLUD1 | AC093673.1 | -0.55619 | 6.27E-58 |
| MAPK10 | AC093673.1 | -0.5314 | 4.02E-52 |
| PPIA | AC093673.1 | 0.668127 | 1.95E-91 |
| JMJD7-PLA2G4B | AC012213.1 | 0.513622 | 3.03E-48 |
| JMJD7-PLA2G4B | PSMA3-AS1 | 0.603593 | 1.68E-70 |
| TYK2 | PSMA3-AS1 | 0.515037 | 1.52E-48 |
| CHMP4A | AC040977.1 | 0.555098 | 1.15E-57 |
| CHMP6 | AC008608.2 | 0.521733 | 5.50E-50 |
