# Supplementary figures and images for: Construction and Validation of a Necroptosis-Related lncRNA Signature in Prognosis and Immune Microenvironment for Glioma
Source: J Oncol. 2022 Aug 27;2022:5681206. doi: 10.1155/2022/5681206 (PMC9440826; doi:10.1155/2022/5681206)

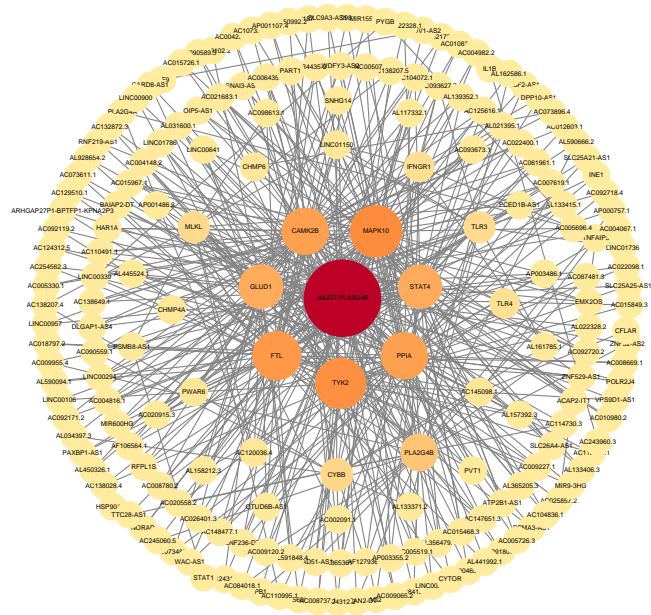

Supplement: Supplementary Materials — Figure S1: association of genes derived from expressional analysis with all lncRNAs with corresponding cor values >0.5. Figure S2: survival analysis of each lncRNA in all glioma patients. Figure S3: among subgroups clustered according to different clinical characteristics, survival differences between high- and low-risk groups. Table S1: prognostic mRNA and their corresponding lncRNA (P < 0.001, cor >0.5) after univariate Cox regression analysis. [file 5681206.f1.zip › FigS1.pdf]

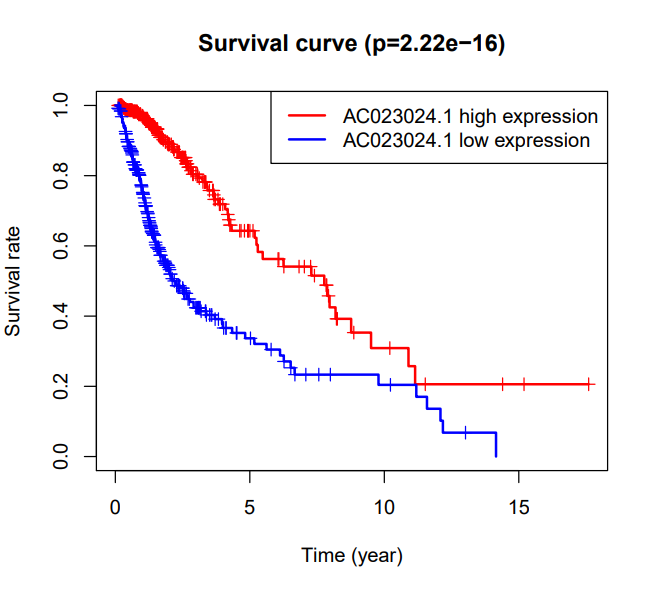

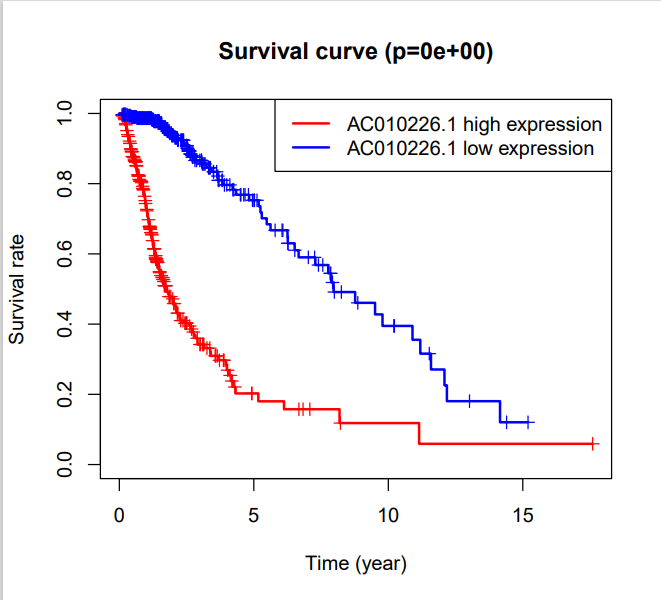

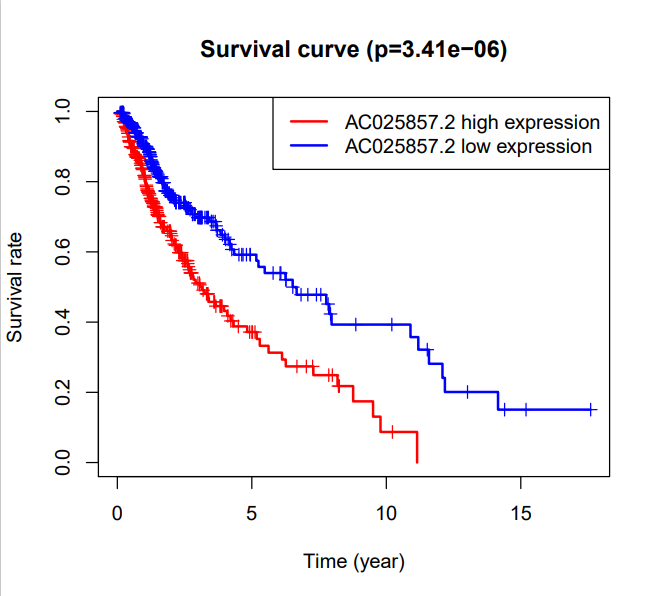

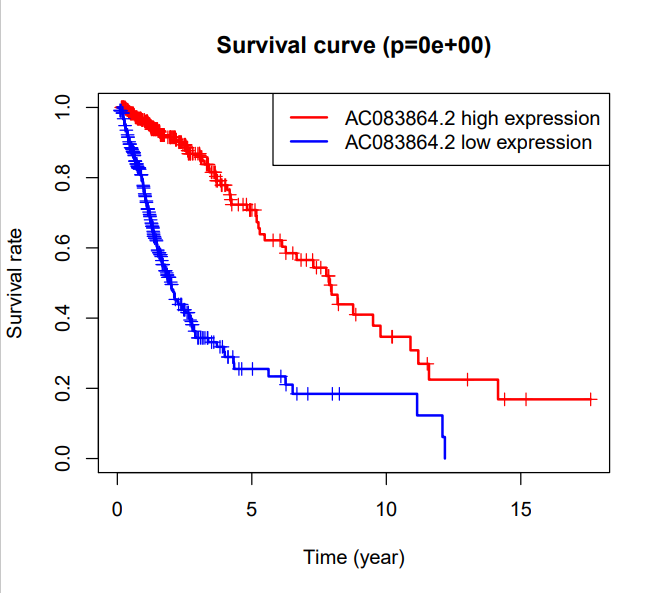

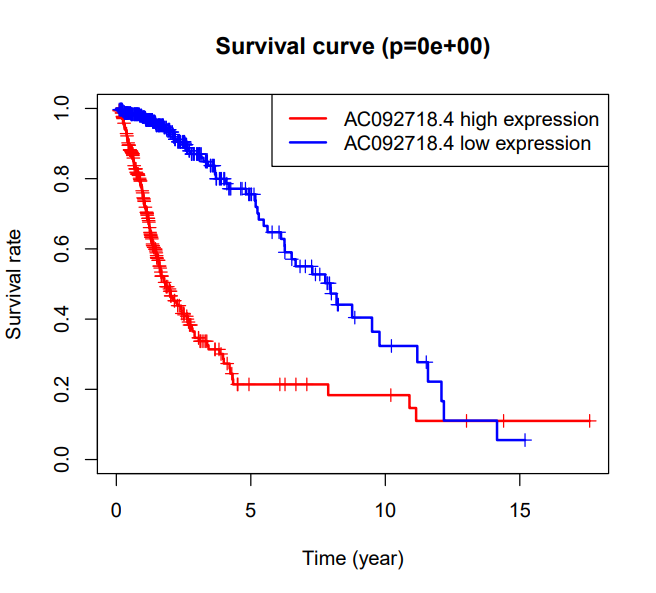

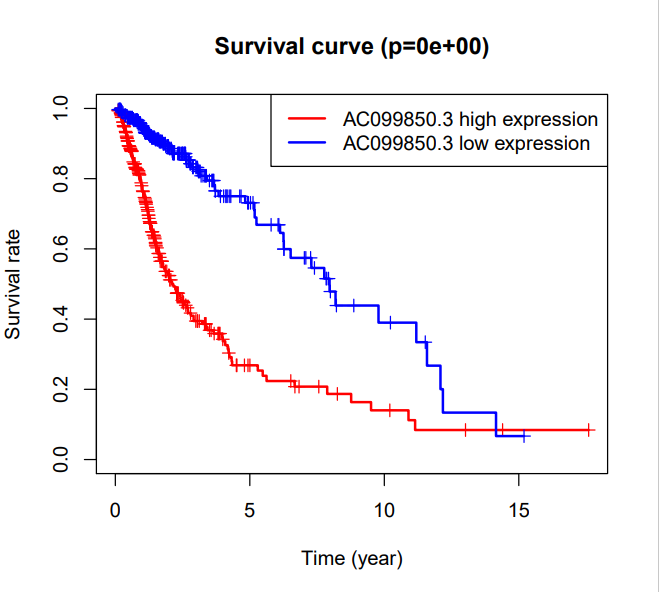

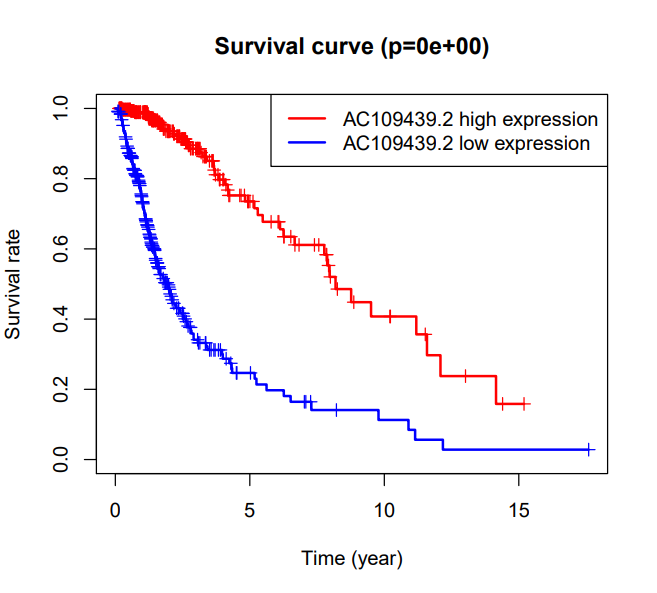

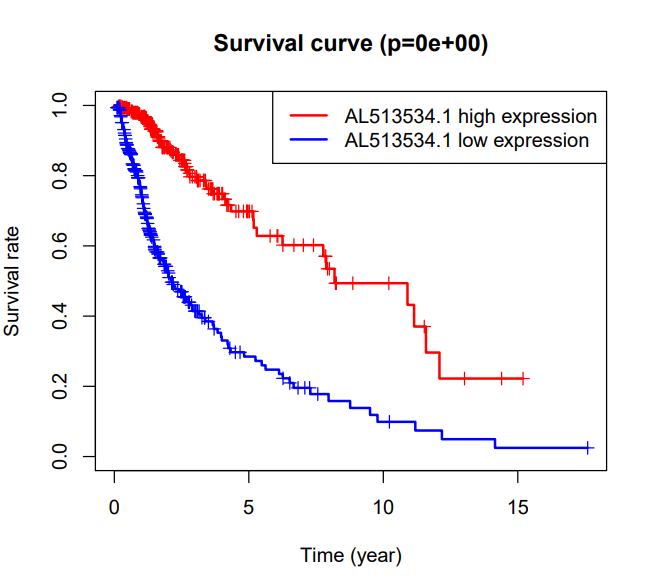

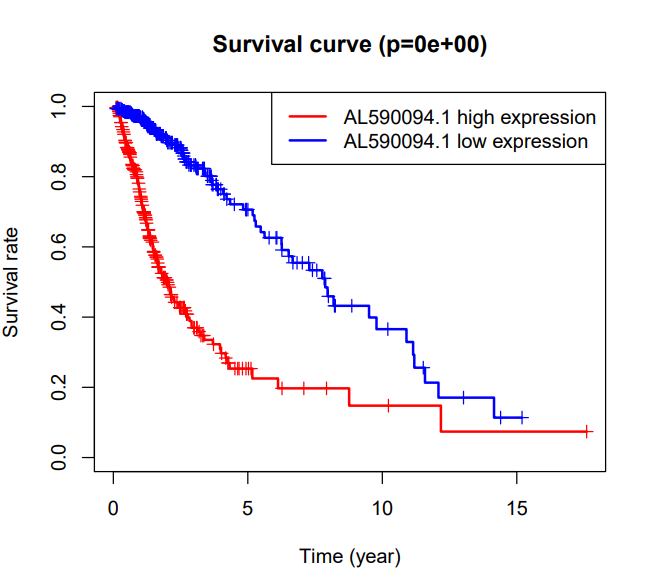

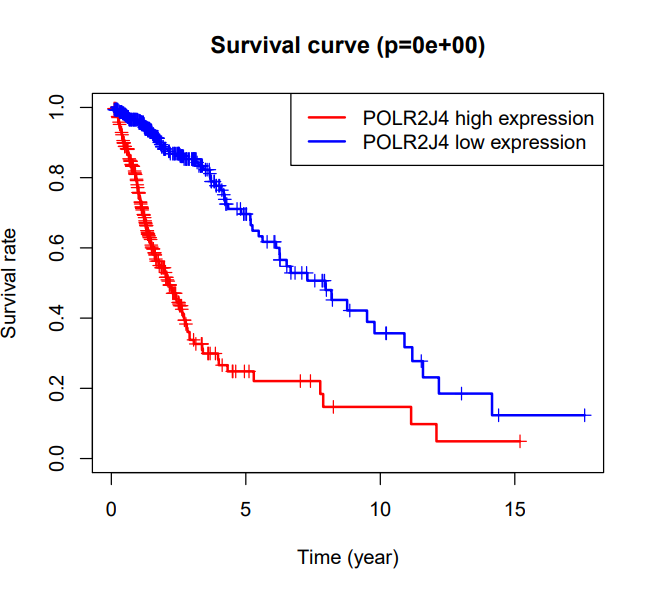

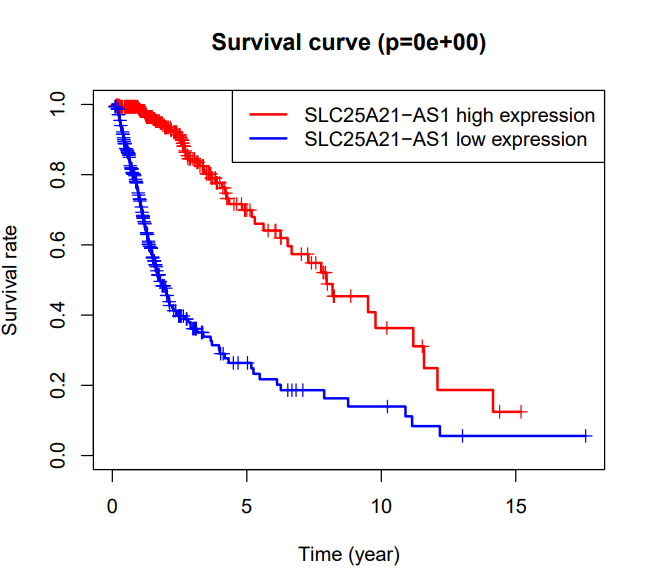

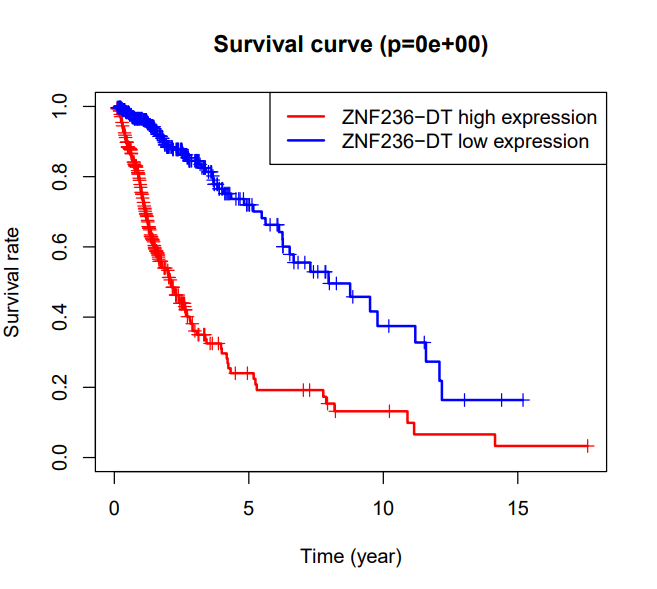

Supplement: Supplementary Materials — Figure S1: association of genes derived from expressional analysis with all lncRNAs with corresponding cor values >0.5. Figure S2: survival analysis of each lncRNA in all glioma patients. Figure S3: among subgroups clustered according to different clinical characteristics, survival differences between high- and low-risk groups. Table S1: prognostic mRNA and their corresponding lncRNA (P < 0.001, cor >0.5) after univariate Cox regression analysis. [file 5681206.f1.zip › FigS2.docx]

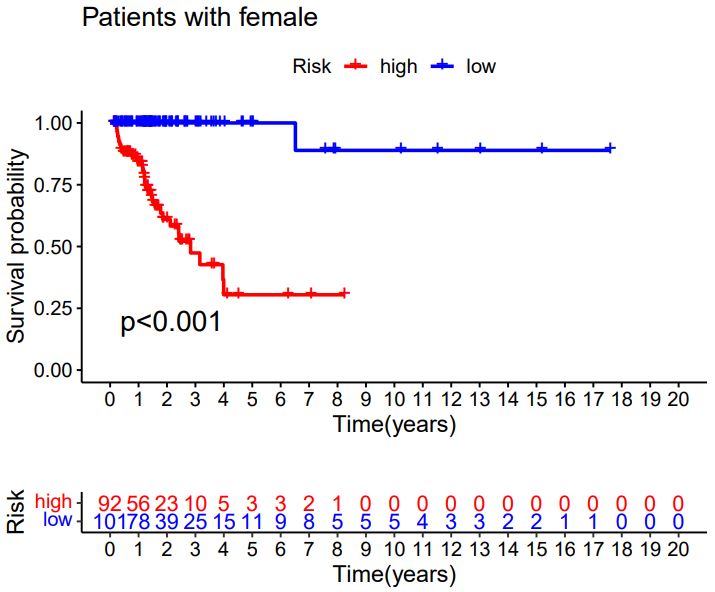

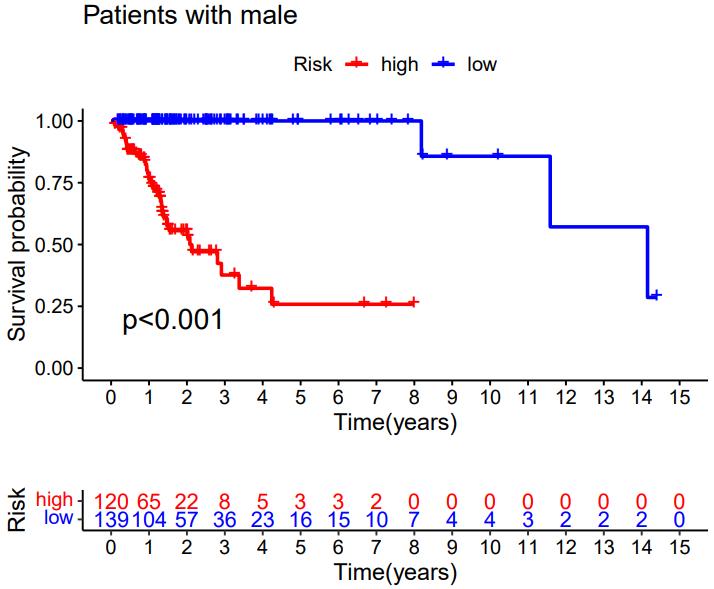

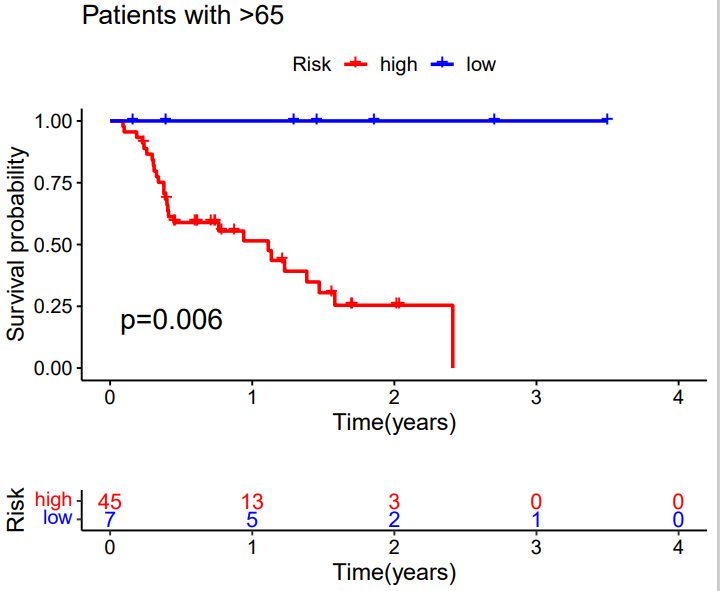

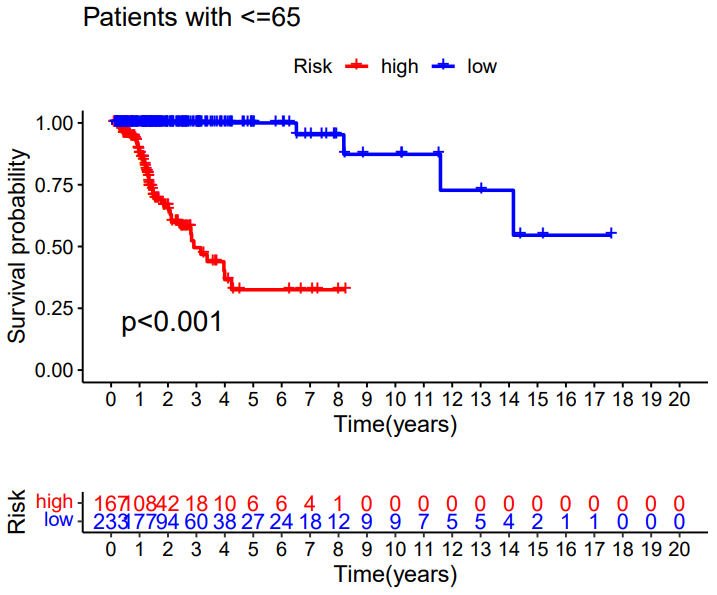

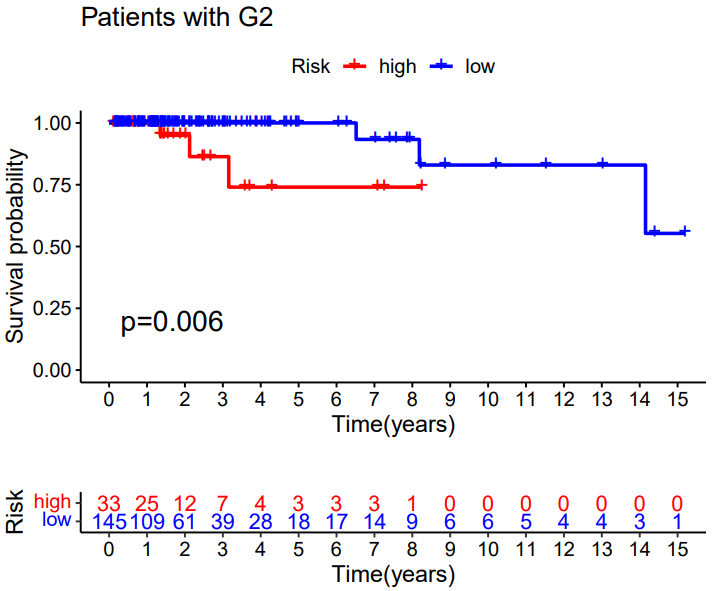

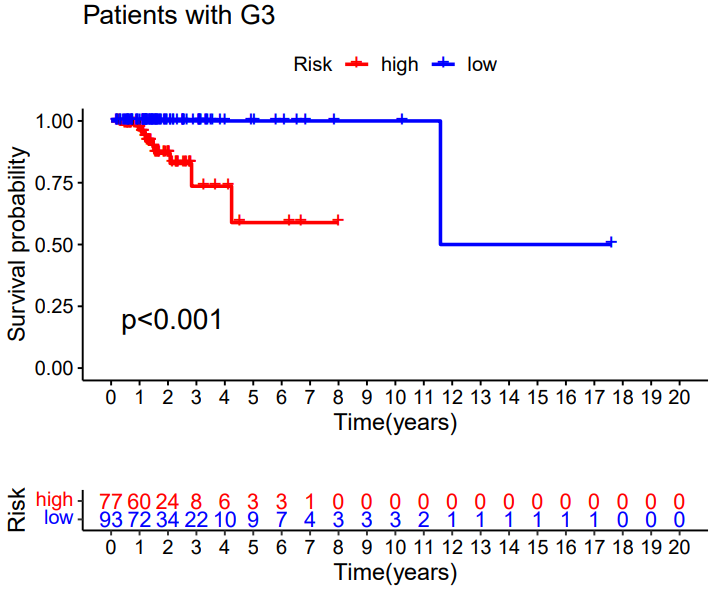

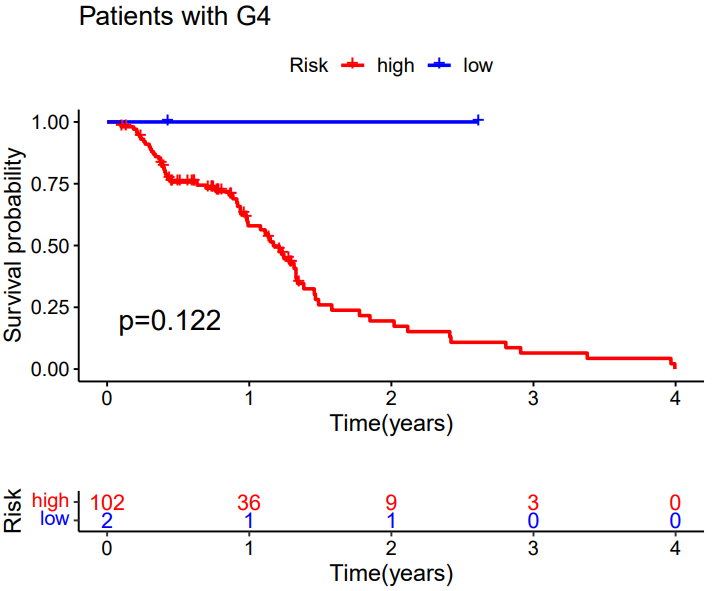

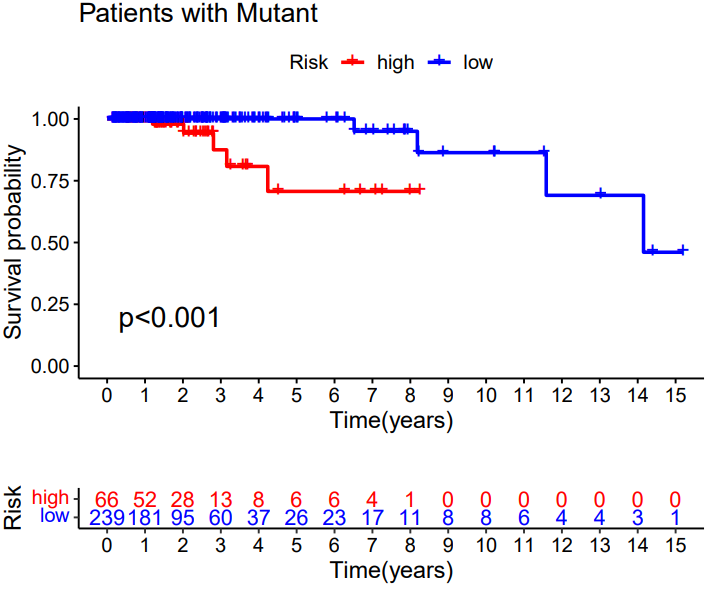

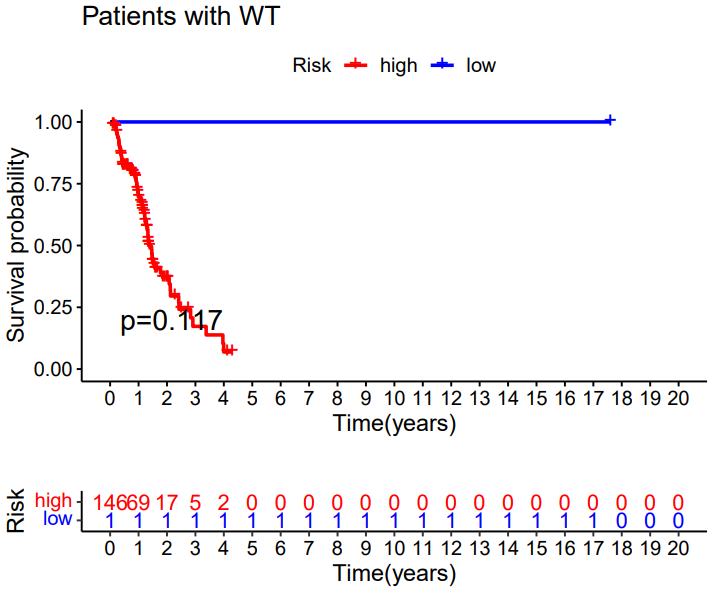

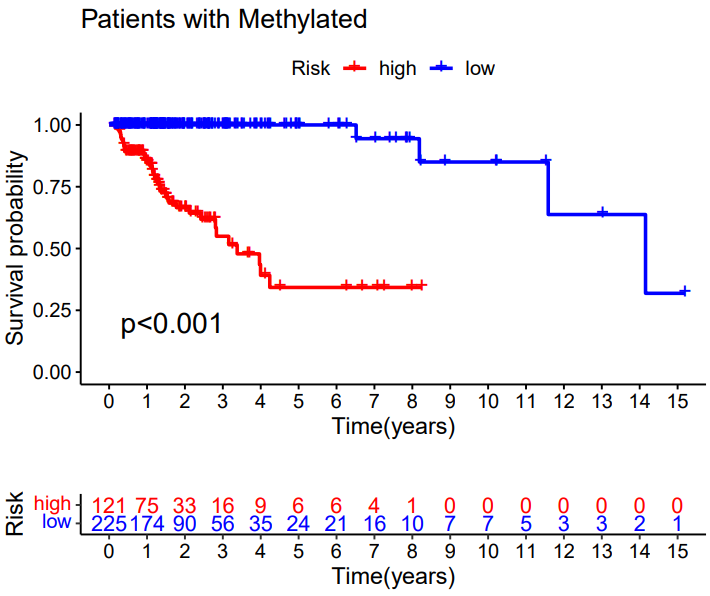

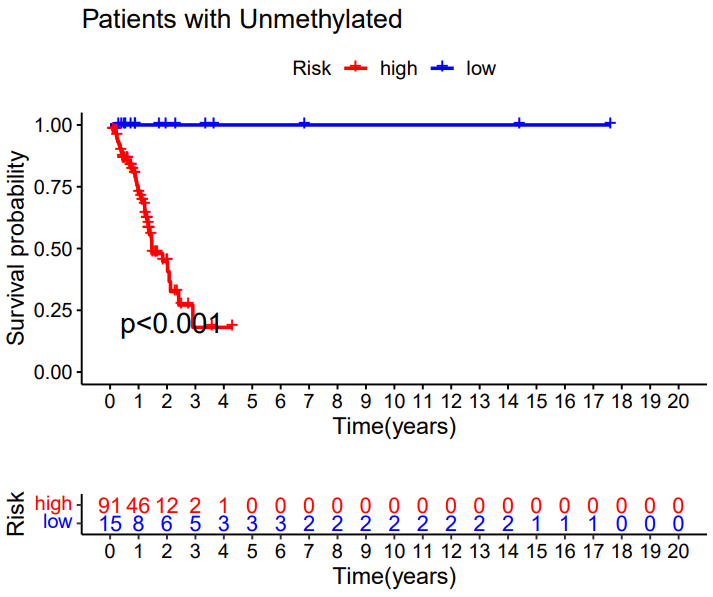

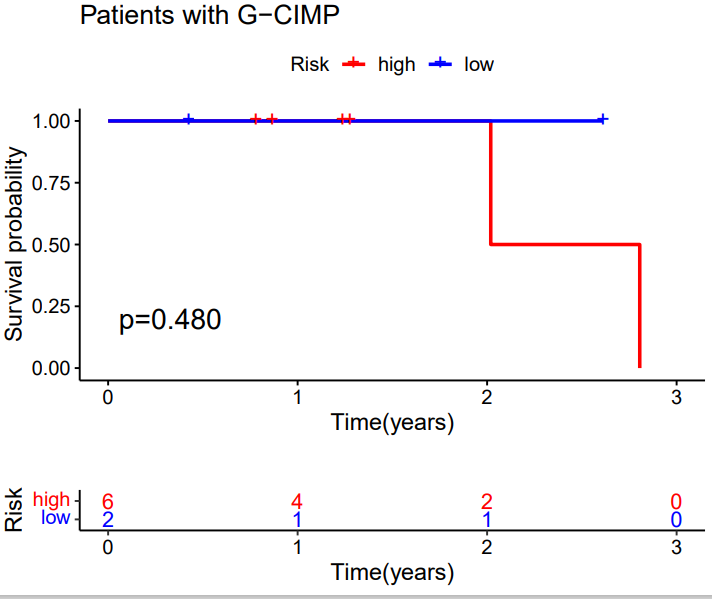

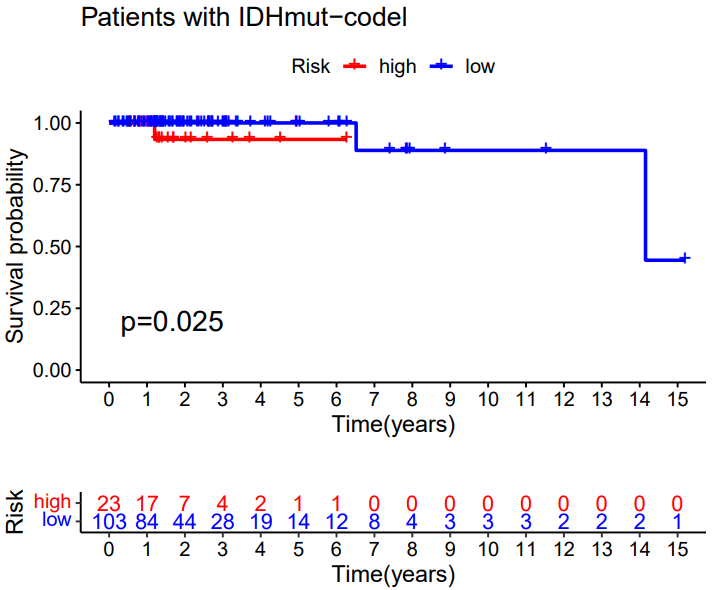

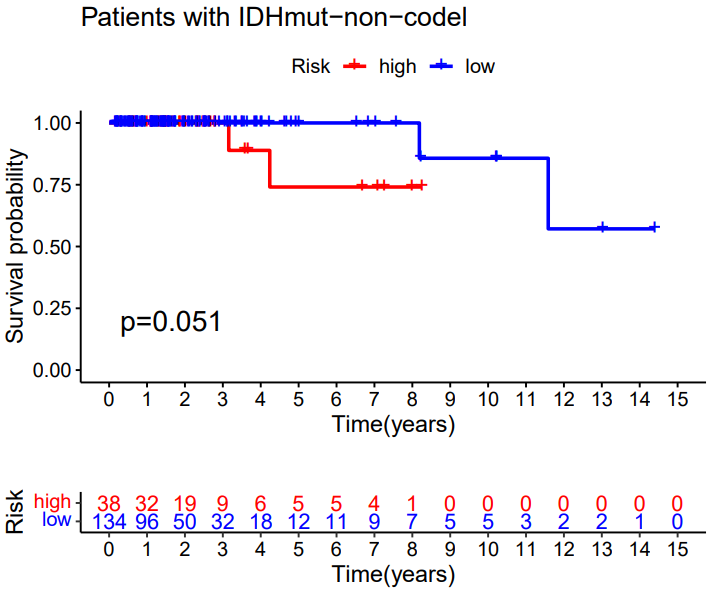

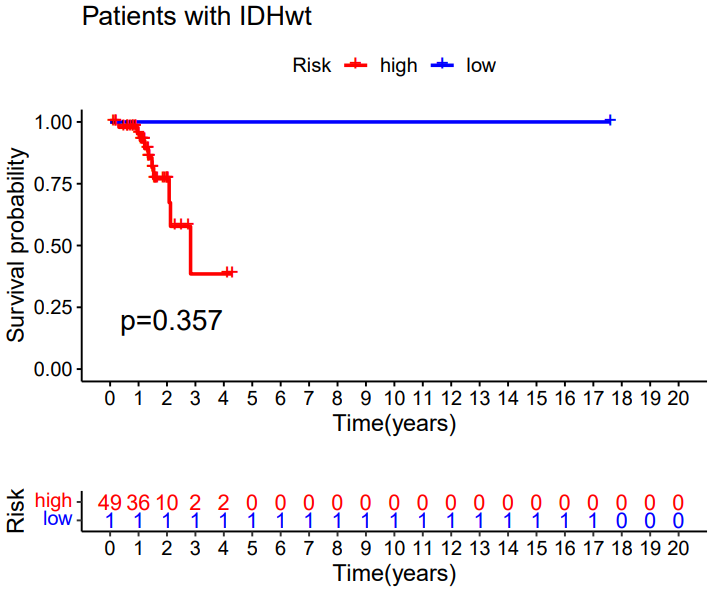

Supplement: Supplementary Materials — Figure S1: association of genes derived from expressional analysis with all lncRNAs with corresponding cor values >0.5. Figure S2: survival analysis of each lncRNA in all glioma patients. Figure S3: among subgroups clustered according to different clinical characteristics, survival differences between high- and low-risk groups. Table S1: prognostic mRNA and their corresponding lncRNA (P < 0.001, cor >0.5) after univariate Cox regression analysis. [file 5681206.f1.zip › FigS3.docx]
